# Supplementary material for: Co-occurrence patterns of malnutrition indicators among children in sub-Saharan Africa
Source: Commun Med (Lond). 2026 Apr 9;6:195. doi: 10.1038/s43856-026-01426-8 (PMC13066028; doi:10.1038/s43856-026-01426-8)
Supplement: Supplementary file 2 — Supplementary Information [file 43856_2026_1426_MOESM2_ESM.pdf]

Supplementary Information: Co-occurrence patterns of malnutrition indicators among children in sub-Saharan Africa

Johannes Seiler [1*], Benjamin Müller [1], Isabel Günther [2,3], Mattias Wetscher [1], Reto Stauffer [1], Nikolaus Umlauf [1], and Kenneth Harttgen [2,3*]

[1]Department of Statistics, University of Innsbruck, Innsbruck, Austria
[2]Development Economics Group, ETH Zurich, Zurich, Switzerland
[3]NADEL Center for Development and Cooperation, ETH Zurich, Zurich, Switzerland
[*]Corresponding authors: e-mail: Johannes.Seiler@uibk.ac.at; Kenneth.Harttgen@nadel.ethz.ch

January 27, 2026

Supplementary Note 1 Overview Supplementary Information

This is the supplementary material for the manuscript *Co-occurrence patterns of malnutrition indicators among children in sub-Saharan Africa* and is organized as follows. Section Supplementary Note 2 discusses further methodological aspects. Section Supplementary Note 3 provides detailed information on the model validation and diagnostic model checks. Section Supplementary Note 4 provides information on the software used. Section Supplementary Note 5 provides additional results, such as the estimated pairwise correlation for different ages.

Supplementary Table 1: Source of additional covariates, including description, periodicity, and source.

Covariate	Description	Periodicity	Source	Reference
Altitude	Elevation in m	Static	NOAA ETOPO	[1, 2]
Conflict	1 if more than five conflict incidents have been reported within buffer, 0 otherwise	Annual	UCDP	[3, 4]
GDP	*log*(real per capita GDP)	Annual	WDI	[5]
Land cover	Land cover classification	Annual	MODIS Land Cover	[6, 7]
Malaria risk	*Plasmodium falciparum* incidence	Annual	Malaria Atlas Project	[8]
Malaria risk	*Plasmodium vivax* incidence	Annual	Malaria Atlas Project	[9]
Malaria risk	*Plasmodium falciparum* temperature suitability	Static	Malaria Atlas Project	[10]
Malaria risk	*Plasmodium vivax* temperature suitability	Static	Malaria Atlas Project	[10]
Malaria risk	*Plasmodium falciparum* endemicity	Static	Malaria Atlas Project	[11]
Malaria risk	*Plasmodium vivax* endemicity	Static	Malaria Atlas Project	[12]
Night-time light	Night-time light	Annual	Night-time light	[13]
NDVI	Normalized difference vegetation index	Monthly	GIMMS, MODIS	[14–16]
Precipitation	Precipitation anomaly	Monthly	ERA5	[17]
Soil	Soil type	Annual	Soilgrids	[18]
Temperature	2 m temperature anomaly	Monthly	ERA5	[17]
Travel Time	Motorized travel time to closest healthcare facility	Static	Malaria Atlas Project	[19]
Travel Time	Walking only travel time to closest healthcare facility	Static	Malaria Atlas Project	[19]
Travel Time	Global travel time to closest city	Static	Malaria Atlas Project	[20]
Waterbody	Distance to body of water	Static	GRG Washington	[21]

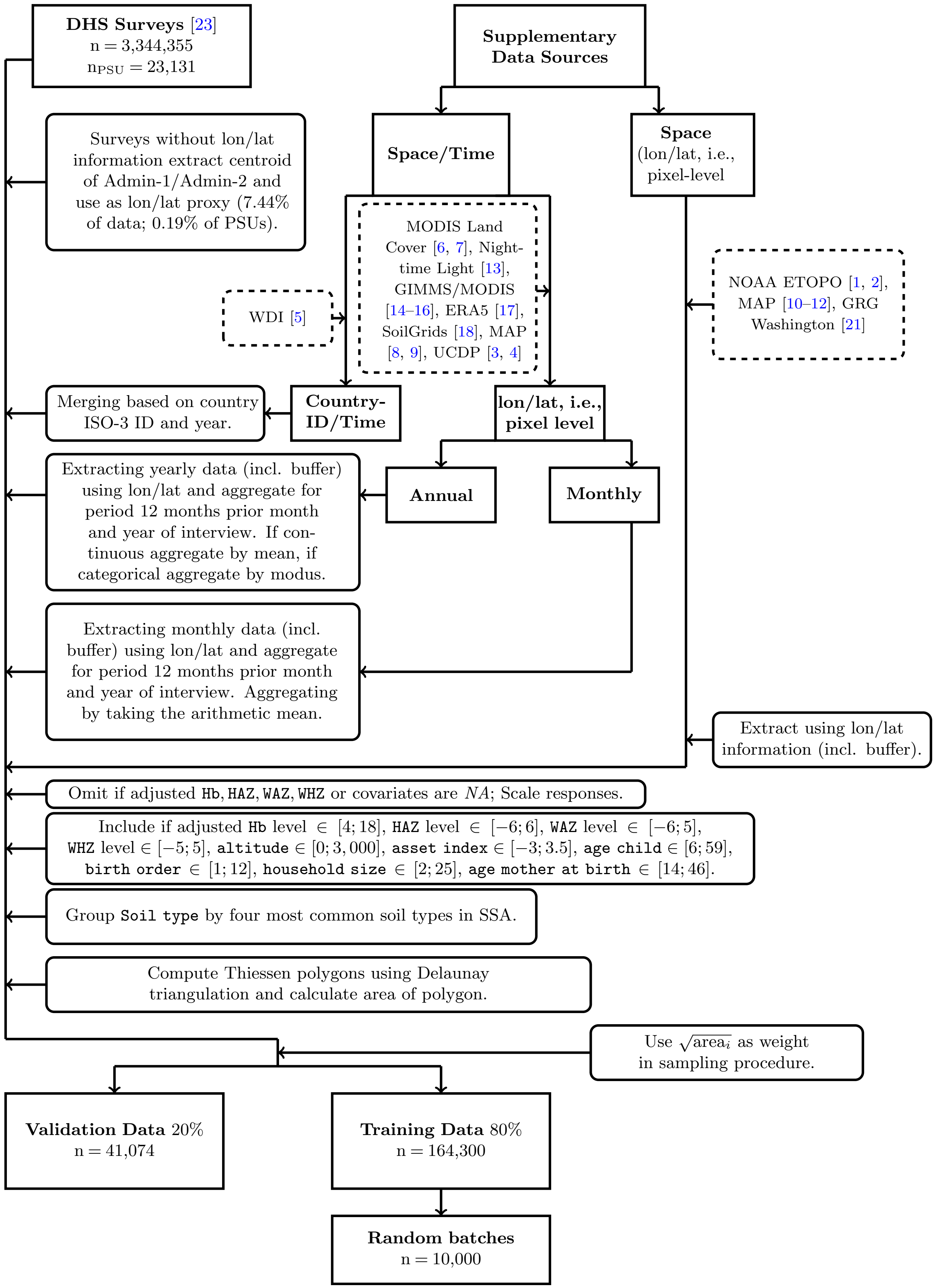

Supplementary Figure 1: Flowchart of data pre-processing. Note that malaria incidence (i.e., `minc12`) was set to 0, if the country is classified as malaria free (e.g., Lesotho [24]).

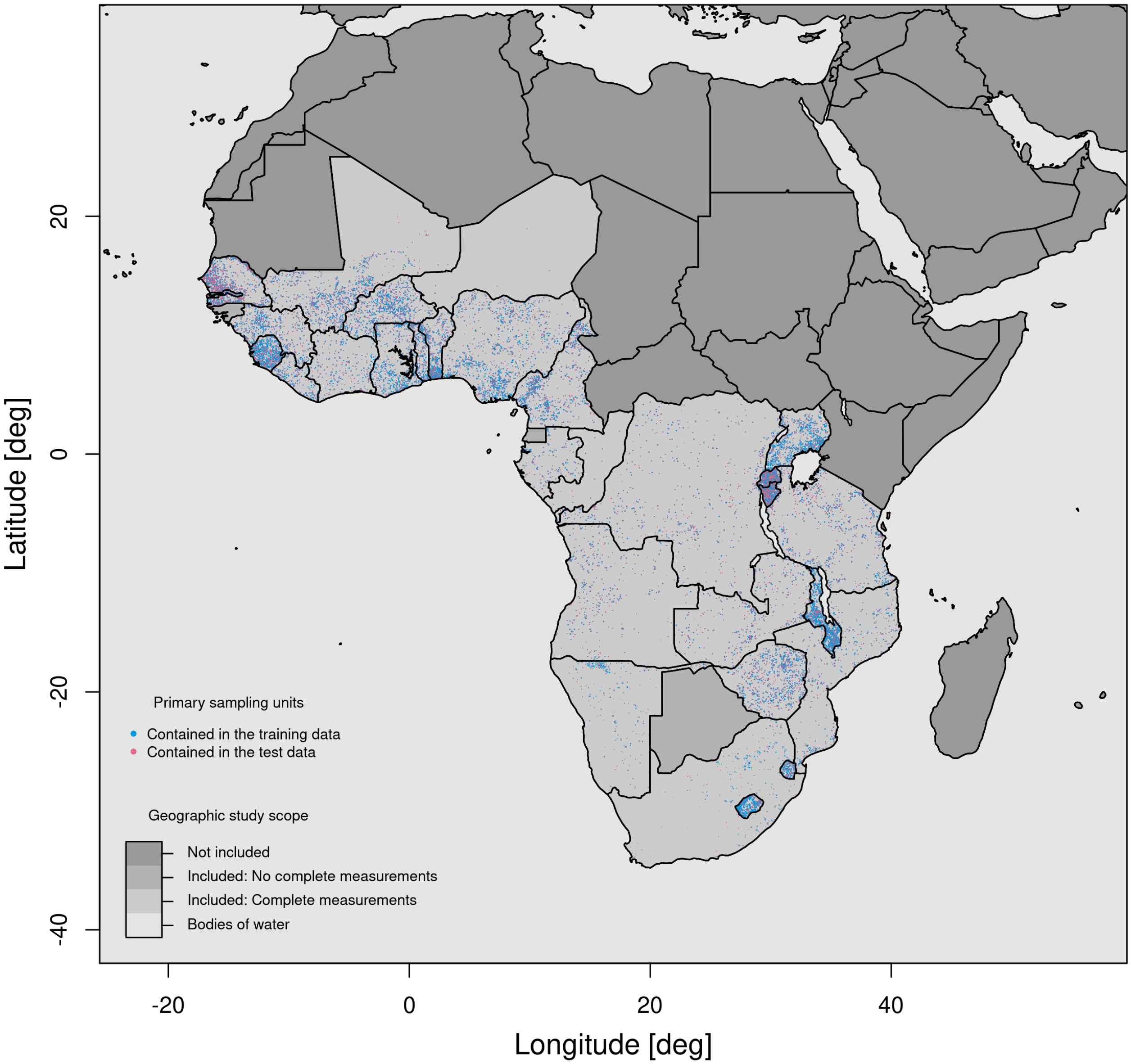

Supplementary Figure 2: **Map of included countries and locations of the primary sampling units.** The gray-scaled colors indicate countries for which measurements of the response variables are available, countries without measurements of the response variables, and countries outside the study area that have been excluded. Colored dots indicate the sample locations included in the training data (n = 164,300) and test data (n = 41,074).

Supplementary Table 2: Pairwise Pearson correlation coefficients between Hb, HAZ, WAZ, and WHZ for each country-year combination.

Country code	Year	$\rho_{\text{Hb HAZ}}$	$\rho_{\text{Hb WAZ}}$	$\rho_{\text{Hb WHZ}}$	$\rho_{\text{HAZ WAZ}}$	$\rho_{\text{HAZ WHZ}}$	$\rho_{\text{WAZ WHZ}}$
AGO	2016	0.09	0.06	-0.00	0.73	-0.01	0.67
BFA	2003	0.05	0.20	0.22	0.59	-0.16	0.70
BFA	2010	0.15	0.19	0.11	0.60	-0.15	0.70
BEN	2006	0.08	0.13	0.07	0.53	-0.35	0.60
BEN	2012	-0.05	0.03	0.08	0.48	-0.43	0.58
BEN	2018	0.12	0.12	0.05	0.72	0.01	0.70
BDI	2010	0.12	0.14	0.08	0.67	-0.03	0.72
BDI	2016	0.16	0.17	0.09	0.74	0.10	0.73
COD	2007	0.04	0.11	0.10	0.65	-0.21	0.60
COD	2014	0.05	0.10	0.08	0.71	-0.13	0.60
COG	2005	0.04	0.11	0.09	0.58	-0.30	0.59
COG	2012	0.07	0.09	0.05	0.68	-0.08	0.67
CIV	2012	0.09	0.12	0.07	0.69	-0.09	0.65
CMR	2004	0.12	0.16	0.10	0.64	-0.14	0.66
CMR	2011	0.09	0.15	0.14	0.70	0.02	0.72
CMR	2018	0.10	0.14	0.09	0.66	-0.11	0.67
GAB	2012	0.10	0.07	0.00	0.65	-0.09	0.69
GHA	2008	0.10	0.13	0.07	0.59	-0.18	0.68
GHA	2014	0.15	0.14	0.05	0.70	0.06	0.75
GMB	2013	0.17	0.13	0.01	0.61	-0.18	0.67
GMB	2019	0.21	0.09	-0.04	0.71	0.16	0.80
GIN	2012	0.13	0.17	0.08	0.65	-0.17	0.63
GIN	2018	0.06	0.06	0.02	0.65	-0.19	0.62
LBR	2019	0.08	0.09	0.04	0.70	-0.04	0.68
LSO	2009	0.05	0.03	0.00	0.61	-0.14	0.69
LSO	2014	0.07	0.07	0.05	0.63	-0.01	0.76
MLI	2006	0.15	0.17	0.08	0.65	-0.13	0.66
MLI	2013	0.19	0.15	0.01	0.72	-0.11	0.60
MLI	2018	0.14	0.15	0.07	0.70	-0.04	0.68
MWI	2010	0.07	0.08	0.05	0.58	-0.24	0.64
MWI	2015	0.03	0.01	-0.01	0.68	-0.06	0.68
MOZ	2011	0.17	0.20	0.09	0.62	-0.19	0.63
NGA	2018	0.19	0.20	0.09	0.76	0.03	0.66
NER	2012	0.11	0.13	0.07	0.66	-0.09	0.68
NAM	2013	0.03	0.00	-0.01	0.64	-0.01	0.76
RWA	2005	0.10	0.13	0.08	0.64	-0.13	0.67
RWA	2010	0.01	0.04	0.06	0.64	-0.11	0.68
RWA	2015	0.07	0.08	0.05	0.66	-0.04	0.71
RWA	2020	0.07	0.05	0.03	0.70	0.06	0.74
SLE	2008	0.09	0.18	0.14	0.64	-0.19	0.63
SLE	2013	0.08	0.03	-0.04	0.63	-0.21	0.62
SLE	2019	0.10	0.09	0.02	0.71	-0.04	0.67
SEN	2005	0.12	0.06	-0.02	0.67	-0.03	0.72
SEN	2011	0.20	0.12	-0.03	0.67	-0.11	0.65
SEN	2013	0.20	0.11	-0.04	0.72	0.05	0.73
SEN	2014	0.21	0.12	-0.03	0.72	0.06	0.73
SEN	2015	0.19	0.10	-0.03	0.72	0.10	0.75
SEN	2016	0.22	0.11	-0.04	0.73	0.11	0.75
SEN	2017	0.18	0.09	-0.04	0.71	0.05	0.73
SWZ	2007	0.06	0.08	0.08	0.60	-0.09	0.73
TGO	2014	0.09	0.08	0.03	0.71	0.03	0.72
TZA	2010	0.06	0.04	0.01	0.62	-0.10	0.71
TZA	2015	0.02	0.04	0.03	0.66	-0.06	0.70
UGA	2006	0.03	0.12	0.16	0.67	-0.04	0.71
UGA	2011	0.11	0.15	0.11	0.68	-0.07	0.67
UGA	2016	0.11	0.15	0.11	0.70	0.00	0.71
ZAF	2016	0.11	0.06	0.01	0.63	0.02	0.78
ZMB	2018	0.10	0.08	0.02	0.61	-0.17	0.67
ZWE	2006	0.04	0.05	0.03	0.57	-0.17	0.70
ZWE	2011	0.03	0.04	0.05	0.62	-0.11	0.70
ZWE	2015	0.08	0.07	0.03	0.61	-0.09	0.73

The country abbreviations correspond to the country-specific ISO3 country codes. *S*ource: Own calculations, based on DHS survey data [22].

Supplementary Note 2 Methods and modeling approach

Generalized additive models (GAM; [25]) and their more flexible extensions, generalized additive models for location, scale, and shape (GAMLSS; [26], which are also known as distributional regession [27–29]), have been utilized in diverse univariate applications across various fields of applied research. These include climate research (see, e.g., [30, 31]) as well as global health topics such as the modeling and mapping of childhood mortality [32–34], and childhood malnutrition [35–38]. This class of regression models is known for characterizing all potential distributional parameters of a given response distribution, as well as for its flexibility in specifying covariate effects. It is particularly useful for accounting for nonlinear effects, capturing group effects beyond a simple parallel shift, or accounting for complex spatio-temporal interactions. Moreover, it exhibits good predictive performance, while the estimated effects remain interpretable. However, extending the GAMLSS framework beyond bivariate responses remains challenging due to computational and theoretical difficulties. Only recently have methodological works [39, 40] enabled the application of the GAMLSS framework to multivariate applications. However, the estimation of multivariate GAMLSS models remains computationally challenging due to time restrictions and memory requirements. Building on these recent developments, we apply a multivariate GAMLSS to a four-dimensional response vector to analyze co-occurrence patterns of childhood malnutrition in sub-Saharan Africa (SSA).

The modeling approach used in this study is similar to the univariate approach described by Seiler and colleagues [38] for modeling anemia prevalence in SSA and South Asia. Further details, along with a schematic illustration of the different modeling steps, can be found in Umlauf and Kneib [41]. The five distinct modeling steps are briefly outlined as follows:

1. **Creating the training and test data set.** Prior to the following modeling steps, the complete data set is divided into a training set containing 80% of the data and a test data set containing the remaining 20%. In this step, due to spatial clustering (see, Roberts et al. [42] for a narrative), the data is not split randomly. Instead, the method described by Gething et al. [11], based on Delaunay triangulation, is used to create the test data set – used only for validation – that is spatially representative for the analyzed countries within SSA. For additional details on the initial pre-processing steps, see *Supplementary Figure* 1.
2. **Estimating a baseline reference model.** To create a basic reference model, an initial model is estimated without including covariates. In this model, the parameters of the mean vector $\boldsymbol{\mu}$ and the variance-covariance matrix $\boldsymbol{\Sigma}$ are estimated, which serve as a reference to benchmark the performance and improvement of the final model.
3. **Identifying informative covariates in each predictor η_k.** In the next step, informative covariates for the multivariate distributional regression model are selected using a boosting algorithm for distributional regression. Details of this algorithm are described in Umlauf et al. [43].
4. **Bayesian estimation of the final model omitting uninformative covariates.** Once the most informative covariates for each distributional parameter η_k are identified, the final model is estimated omitting uninformative covariates using Markov Chain Monte Carlo (MCMC) sampling.
5. **Model validation.** To evaluate the predictive performance of the final models and to analyze the model calibration on in-sample and out-of-sample data, graphical methods, the log-likelihood, the Akaike Information Criterion (*AIC*; [44]), and the logarithmic score (*logS*; [45]) are inspected and compared to the reference model. In addition, a cross-validation routine is applied to investigate potential sparseness problems associated with the data.

Before elaborating on these five modeling steps in Supplementary Note 2.2 a brief overview of the GAMLSS framework, and in particular the incorporation of multivariate response vectors will be given.

Supplementary Note 2.1 Methodological background

Distributional regression framework GAMLSS [26] is an extension of the well-known GAM [25] that allows modeling of any arbitrary parametric response distribution (e.g., beta distribution, gamma distribution,

multivariate Gaussian distribution), even beyond the exponential family. Accordingly, all parameters of a given response distribution can be linked to an additive predictor and thus can be modeled based on incorporating covariates. Specifically, this allows to specify a multivariate Gaussian distribution that can be facilitated to study and identify co-occurrence patterns, and common risk factors jointly associated with various forms of malnutrition. Accordingly, using this approach allows to relate all parameters of the multivariate Gaussian response distribution to socio-economic, environmental, and climatic covariates. Moreover, by incorporating regression effects for all parameters of the multivariate response distribution, each of the K parameters can be associated with a distinct predictor η_k. This yields the following structure:

$$\begin{aligned}\mathbf{y}|\mathbf{x},\mathbf{z} \sim \mathcal{D}_y\big(\theta_1(\mathbf{x},\mathbf{z}) = h_1^{-1}(\eta_1(\mathbf{x},\mathbf{z})), \theta_2(\mathbf{x},\mathbf{z}) = h_2^{-1}(\eta_2(\mathbf{x},\mathbf{z})), ...,\\ \theta_K(\mathbf{x},\mathbf{z}) = h_K^{-1}(\eta_K(\mathbf{x},\mathbf{z}))\big).\end{aligned} \tag{1}$$

In Equation 1, $\mathcal{D}_y$ specifies the parametric distribution family of the response $\mathbf{y}$ (e.g., multivariate Gaussian distribution, i.e., $\mathbf{y} \sim \mathcal{N}(\boldsymbol{\mu}, \boldsymbol{\Sigma})$, with $\boldsymbol{\mu} = \mathrm{E}(\mathbf{y})$ the expectation of $\mathbf{y}$ and $\boldsymbol{\Sigma}$ the variance-covariance matrix); $\theta_1(\mathbf{x},\mathbf{z}), ..., \theta_K(\mathbf{x},\mathbf{y})$ the parameters of the distribution $\mathcal{D}_y$; $\mathbf{x}$ the vectors of parametric model terms, i.e., the covariates that enter the model linearly; and $\mathbf{z}$ the vectors of explanatory covariates entering the model as non-parametric (smooth) functions. The parameters θ_k ($k = 1, ..., K$) of the distribution $\mathcal{D}_y$ are related to additive predictors $\eta_k \equiv \eta_k(\mathbf{x},\mathbf{z})$ with separate suitable link functions h_k^{-1} to ensure the potential restrictions of the parameter space. Thus, the predictor η_k of the distribution parameter θ_k has the following form,

$$\eta_k = \eta_k(\mathbf{x}, \boldsymbol{\gamma}; \mathbf{z}, \boldsymbol{\beta}_k) = \mathbf{x}^\top \boldsymbol{\gamma} + f_1(\mathbf{z}, \boldsymbol{\beta}_{1k}) + \ldots + f_{19}(\mathbf{z}, \boldsymbol{\beta}_{19k}), \tag{2}$$

and may include, among others, various model terms, such as linear terms, non-linear smooth terms, or complex interactions between covariates. Often, the predictor η_k is also referred to as a structured additive predictor (STAR; [46]). Two common methods can be incorporated: thin-plate regression splines [47] to model univariate effects assuming potentially non-linear, smooth functions, and tensor product splines [48] to capture interactions between variables, such as space and time or space and age.

Cholesky-based parameterization of the variance-covariance matrix $\boldsymbol{\Sigma}$ Following the approach proposed by Pourahmadi [49] and adapted to distributional regression models by Muschinski et al. [39], the variance-covariance matrix $\boldsymbol{\Sigma}$ is re-parameterized by a (modified) Cholesky decomposition. This ensures positive definiteness of all distributional parameters associated with the covariance structure. Accordingly, all parameters θ_k of $\mathcal{D}_y$ can be estimated using standard regression routines for distributional regression, such as, the batch-wise backfitting algorithm proposed by Umlauf et al. [43] – capable of automated variable selection – or classical Bayesian inference based on MCMC sampling.

More precisely, following Muschinski et al. [39], the response $\mathbf{y}$ is a vector of length M (here $M = 4$), i.e., $\mathbf{y} = (y_{i1}, \ldots, y_{iM})^\top$, which is assumed to be linked to an M-dimensional Gaussian distribution, i.e., $\mathbf{y} \sim \mathcal{N}(\boldsymbol{\mu}, \boldsymbol{\Sigma})$ that has the following density function:

$$f(\mathbf{y}|\boldsymbol{\mu}, \boldsymbol{\Sigma}) = \frac{1}{\sqrt{(2\pi)^M |\boldsymbol{\Sigma}|}} \exp\left\{-\frac{1}{2}(\mathbf{y}-\boldsymbol{\mu})^\top \boldsymbol{\Sigma}^{-1}(\mathbf{y}-\boldsymbol{\mu})\right\}, \tag{3}$$

where, $\boldsymbol{\mu} = \mathrm{E}(\mathbf{y})$, corresponds to the vector of expected values of the response vector $\mathbf{y}$. Note that $f(\mathbf{y}|\boldsymbol{\mu}, \boldsymbol{\Sigma})$ exists only if $\boldsymbol{\Sigma}$ is positive definite. This is usually achieved by using parameterizations of $\boldsymbol{\Sigma}$ that guarantees positive definiteness, such as the approach based on a (modified) Cholesky decomposition – i.e., decomposing $\boldsymbol{\Sigma}$ into a lower triangular matrix $\mathbf{L}$, such that $\boldsymbol{\Sigma} = \mathbf{L}\mathbf{L}^\top$ holds. Estimation of this type of model relies on the full infrastructure of the R package **bamlss** [43, 50, 51], which provides Frequentist as well as Bayesian estimation routines allowing for the estimation of complex distributional regression models.

Supplementary Note 2.2 Modeling approach

Creating the training and test data set Geo-referenced survey data can be merged with other geo-located data sources that may serve as proxies for, e.g., infectious diseases or environmental and climatic factors associated with child malnutrition. Although the merging process – based on positional information – and

the subsequent splitting into training and test data sets are straightforward, they must be performed with great caution due to several potential pitfalls. For instance, different remote sensing data may have varying projections, spatial resolutions, or temporal granularities. More critically, as Roberts et al. [42] point out, in the presence of spatial or spatio-temporal clustering, dependency structures often exist that preclude arbitrarily dividing the data set into training and validation subsets. Such naive partitioning can lead to estimation errors and unreliable error estimates, resulting in overly optimistic model performance. A simple yet effective remedy is to create irregular blocks (e.g., by constructing Thiessen polygons around each sampled location i) based on the spatial distribution of the data. The test data set can then be drawn from these blocks, using the area of the polygon surrounding location i (or a monotonic transformation such as $\sqrt{\text{area}_i}$) as the sampling probability (see Roberts et al. [42] for a detailed discussion). Using this approach, the complete data set is divided into a training set – containing approximately 80% of the data – and a test set – containing approximately 20% – by assigning each location i a sampling probability and drawing (without replacement) the test data set. The training data set consists of the remaining observations not included in the test set. See *Supplementary Figure* 1 for a visual overview of the preprocessing steps and *Supplementary Figure* 2 for a reference map showing the primary sampling units (PSUs). Applications of this method, along with additional procedural details, can be found in Gething et al. [11] and Seiler et al. [38].

Estimating a baseline reference model To establish a baseline model for comparison, an initial model is estimated by omitting all covariates from each predictor η_k. In this multivariate Gaussian setting, only the parameters of the mean vector $\boldsymbol{\mu}$ and the variance-covariance matrix $\boldsymbol{\Sigma}$ are estimated, without incorporating any covariate information. Accordingly, the baseline model provides estimates for the four location parameters, the four scale parameters, and six correlation parameters omitting any covariate information, and provides a rigorous benchmark for assessing the performance improvements and accuracy gains of the final model.

Identifying informative covariates in each predictor η_k

The approach proposed by Muschinski et al. [39] for estimating multivariate Gaussian responses is embedded within the framework of distributional regression models. Accordingly, the batchwise backfitting algorithm introduced by Umlauf et al. [43] can be employed to identify informative covariates. This estimation procedure builds on the backfitting strategy implemented by Umlauf et al. [50], applying an iterative weighted least squares (IWLS; [52]) update at each step.

Starting from the full model specified in Equation 4 – which includes all candidate covariates in each predictor η_k – the algorithm performs iterative updates until a stopping criterion is met. Specifically, when the relative change in the out-of-sample log-likelihood falls below a predefined threshold. Only those covariates in each predictor η_k are updated that are identified to have the largest contribution to the individual log-likelihood. The full predictor η_k is specified as follows:

$$
\begin{aligned}
\eta_k = {} & \texttt{gf} \cdot \gamma_1 + \texttt{vaccine} \cdot \gamma_2 + \texttt{education} \cdot \gamma_3 + \texttt{conf25} \cdot \gamma_4 + \texttt{conf50} \cdot \gamma_5 + \\
& \texttt{conf100} \cdot \gamma_6 + \texttt{lc12} \cdot \gamma_7 + \texttt{soil2} \cdot \gamma_8 + \texttt{nl20\_12} \cdot \gamma_9 + f_1(\texttt{ai}) + f_2(\texttt{bord}) + \\
& f_3(\texttt{cage}) + f_4(\texttt{hhs}) + f_5(\texttt{l\_gpd}) + f_6(\texttt{magebirth}) + f_7(\texttt{altitude}) + \\
& f_8(\texttt{l\_distance}) + f_9(\texttt{l\_ttcity}) + f_{10}(\texttt{l\_ttmotor}) + f_{11}(\texttt{l\_ttwalk}) + \\
& f_{12}(\texttt{minc12}) + f_{13}(\texttt{ndvi12}) + f_{14}(\texttt{pre12}) + f_{15}(\texttt{iyear}) + f_{16}(\texttt{t2m12}) + \\
& f_{17}(\texttt{x}, \texttt{y}) + f_{18}(\texttt{x}, \texttt{y}, \texttt{cage}) + f_{19}(\texttt{x}, \texttt{y}, \texttt{iyear}).
\end{aligned} \tag{4}
$$

See *Supplementary Table* 3 for a comprehensive overview of all included covariates and model specification.

Compared to the classical backfitting algorithm, which calculates updates of the model terms in each iteration based on the entire training data set, the batchwise backfitting method [43] draws small random batches (i.e., n=10,000 in this application) of the training data set for each iteration, which are used to calculate the updates of the model terms. Correspondingly, the training data set used in this application uses T=1,500 randomly drawn batches $\mathbf{b}_1, \ldots, \mathbf{b}_T$ sampled with replacement from the training data set. In each iteration, $t = 1, \ldots, T$, the algorithm calculates potential updates for all model terms on the current batch $\mathbf{i} = \mathbf{b}_t$ and selects the model term that gives the best improvement in the out-of-sample log-likelihood (i.e., the log-likelihood

improvement of the next batch $\tilde{\mathbf{i}} = \mathbf{b}_{t+1}$). Using two batches in each iteration increases the stability of the estimates, since the second batch $\tilde{\mathbf{i}}$ can be considered mimicking unseen out-of-sample data. By using only small batches – instead of the full training data set – in each iteration, the computational cost of computing the updates is drastically reduced. In addition, a stochastic updating step of the following form is used for the $jk-$th model term:

$$\boldsymbol{\beta}_{jk}^{[t+1]} = (1 - \nu) \cdot \boldsymbol{\beta}_{jk}^{[t]} + \nu \cdot \boldsymbol{\beta}_{[i],jk}. \quad (5)$$

Here, ν (i.e., $\nu = 0.1$) is a step length parameter that controls the size of the update of the current estimate $\boldsymbol{\beta}_{jk}^{[t]}$ towards $\boldsymbol{\beta}_{jk}^{[t+1]}$. In each iteration of the algorithm, the randomly selected blocks $\mathbf{i}$ and $\tilde{\mathbf{i}}$ from the training data set are used by the algorithm to iterate through all distributional parameters (indexed by k) and, for each k, through all model terms (indexed by j). After a pre-specified number of iterations (i.e., here T=1,500), model terms that have not been updated, and thus the estimated effect is still zero, are removed before the final model. See Umlauf et al. [43] for more details on the algorithm, and Seiler et al. [38] for a detailed presentation of the batchwise backfitting approach for univariate responses, which can be easily extended to vectors of multivariate responses. In the next step, MCMC is used to estimate the model of Equation 4, omitting uninformative covariates and to estimate the regression coefficients $\boldsymbol{\gamma}_{jk}$ and $\boldsymbol{\beta}_{jk}$.

Bayesian estimation of the final model omitting uninformative covariates

The final model is estimated using a Bayesian approach (i.e., MCMC sampling), as Frequentist approaches do not ensure valid inference in the context of distributional regression – a necessity for making informed recommendations in applications. Accordingly, in the final model, the set of predictors η_k in Equation 4 includes only informative covariates. See Figure 1 of the manuscript for a comprehensive overview of which covariates have been selected in each predictor η_k. Analogous to the previous step, each predictor η_k corresponding to the distributional parameter $\theta_k(\mathbf{x}, \mathbf{z})$ is linked to a structured additive predictor via a common link function, which is assumed to be monotonic and twice differentiable [50], i.e.,

$$\theta_k(\mathbf{x}, \mathbf{z}) = h_k(\eta_k^{\theta_k}). \quad (6)$$

An important consideration in Bayesian estimation is the specification of prior distributions. For linear effects, a common choice is to use weakly informative normal priors for $\boldsymbol{\gamma}$, i.e., $p_{jk}(\gamma_{jk}|y) \sim \mathcal{N}(\mathbf{n}, \mathbf{N})$, where $\mathbf{n}$ denotes the prior mean and $\mathbf{N}$ denotes the prior covariance matrix. To make the prior uninformative, one typically sets large values for the variances in $\mathbf{N}$ – or equivalently, allows the precision matrix $\mathbf{N}^{-1}$ to approach zero – thereby flattening the distribution. While such a prior approximates a uniform distribution in the limit, it remains a true Gaussian distribution with a very large spread. Further, it is also important to clarify whether the prior is parameterized in terms of the standard deviation σ or the variance σ^2; the present approach uses σ^2, yielding a prior that is weakly informative but effectively nonrestrictive. This ensures an appropriate prior that does not impose strong constraints on the linear effects. For further discussion of prior selection for linear components, see e.g., Umlauf et al.[50] or Fahrmeir et al. [46].

For non-linear effects, typically modeled using a basis function approach, a multivariate normal prior is assigned to the basis function $\boldsymbol{\beta}_{jk}$, with a precision matrix that is specific to each model term (see, e.g., [50]). Accordingly, the prior $p_{jk}(\cdot)$ of $\boldsymbol{\beta}_{jk}$ takes the form of a multivariate normal kernel, given by: $d_{\boldsymbol{\beta}_{jk}}(\boldsymbol{\beta}_{jk}|\boldsymbol{\tau}_{jk}, \boldsymbol{\alpha}_{\boldsymbol{\beta}_{jk}}) \propto |\boldsymbol{P}_{jk}(\boldsymbol{\tau}_{jk})|^{\frac{1}{2}}\exp\Big(-\frac{1}{2}\boldsymbol{\beta}_{jk}^{\top}\boldsymbol{P}_{jk}(\boldsymbol{\tau}_{jk})\boldsymbol{\beta}_{jk}\Big)$. By specifying the penalty matrices $\boldsymbol{\alpha}_{\boldsymbol{\beta}_{jk}} = \{\boldsymbol{K}_{1jk}, ..., \boldsymbol{K}_{Ljk}\}$ for the basis function coefficients $\boldsymbol{\beta}_{jk}$ the precision matrix, e.g., for univariate splines $\boldsymbol{P}_{jk}(\boldsymbol{\tau}_{jk}) = \tau_{1jk}^{-2}\mathbf{K}_{1jk}$, can be obtained, where the penalty matrices are specific to the chosen smooth term specification. For the smoothing variances $\boldsymbol{\tau}_{jk}$ an inverse gamma distribution is assigned to each $\boldsymbol{\tau}_{jk} = (\tau_{1_{jk}}, ..., \tau_{L_{jk}})^{\top}$. These smoothing variances, which are also sampled from the posterior distribution, control the level of smoothness and overfitting, analogous to the Frequentist smoothing parameter. The primary motivation for using a Bayesian model is not to incorporate prior knowledge for model improvement, but to achieve valid inference – something that is nearly impossible to obtain using a Frequentist approach for distributional regression models. For further details on prior choices for non-linear effects, see Umlauf et al. [50]. Generic multivariate normal priors for smooth functions, with a precision matrix $\boldsymbol{P}_{jk}$ specific to the basis function $\boldsymbol{\beta}_{jk}$ have shown high efficiency and robustness in various applications (see, e.g., [29, 38, 41, 51, 53, 54]).

The main challenge of applying Bayesian MCMC sampling in this multivariate Gaussian regression is the – in this context – very large size of the training data set with more than 160,000 observations. Using the standard MCMC setup from the R-package **bamlss** [50, 51, 55], with a modest number of 6,000 iterations, requires approximately 140 hours on the available HPC infrastructure. To address this and to ensure a satisfactory mixing of the resulting samples, we run 20 parallel MCMC chains, each with 6,000 iterations, a burn-in of 1,000, and thinning of 200. This parallel setup enables efficient estimation via Bayesian simulation techniques while retaining desirable sampling properties. Re-estimating the final model with this approach ensures valid inference, which is crucial for policy-relevant recommendations.

Model validation

The performance and adequacy of the estimated multivariate Gaussian models are assessed by evaluating several validation routines on both the in-sample and out-of-sample data sets. Specifically, the normalized quantile residuals [56], the PIT histogram [57], the *AIC* [44], and appropriate proper scoring rules [58] are used to assess the adequacy of the estimated models and to discriminate between the baseline and final models. These three diagnostic and validation checks are summarized below:

(i) The *Quantile residuals* provide a simple yet useful way to assess whether a model is correctly specified. In univariate settings, the quantile residuals are defined as $\hat{r}_i = \Phi^{-1}\big(\mathcal{F}(y_i|\hat{\boldsymbol{\theta}}_i)\big)$, where Φ^{-1} is the inverse cumulative distribution function of the standard normal distribution [56]. However, the concept of randomized quantile residuals cannot easily be applied to multivariate distributions as there is no analog of the probability integral transform for multivariate distributions [59]. Therefore, quantile residuals can only be applied to the individual marginal distributions separately, allowing the investigation of potential deviations in the marginal specification of continuous outcomes. A model is considered to be well specified at the marginal level if all marginal quantile residuals only show minor deviations from the bisecting line and thus follow approximately a standard normal distribution. (ii) The *Probability integral transform (PIT) histogram* [57] is used to evaluate the calibration of a model based on the marginal quantile residuals. A model is considered well-calibrated at the marginal level when the PIT values u_i follow approximately a uniform distribution on the interval $[0, 1]$. Ensuring calibration at the marginal level and investigating potential miscalibration at the marginal level is crucial for ruling out compensation effects and ensuring the model is well-calibrated [60, 61]. (iii) The *AIC* [44] is defined as $AIC = 2K - log(\hat{L})$, where K is the number of estimated parameters and $\hat{L}$ is the maximum of the likelihood function. The *AIC* is used to assess model fit, providing a trade-off between model complexity and the fit to the data. Thus, it helps in selecting a parsimonious yet accurate model and in assessing the improvement of the final model over the baseline reference model. (iv) In addition, the *logS*, defined as $logS(F, y) = -log(f(y))$ for univariate response distributions [58], can easily be extended to multivariate response vectors. Thus, the *logS* for multivariate responses is defined as follows: $logS(F, \mathbf{y}) = -log(f(\mathbf{y}))$ [58, 62, 63]. This allows the quality of probabilistic prediction to be compared between different competing models, with lower values indicating better prediction performance.

Both the *AIC* and *logS* of the final model are compared to those of a baseline reference model to quantify the improvement. In addition, all diagnostic checks were performed on both the in-sample and out-of-sample data sets. Together with the cross-validation routine (see Section Robustness check for potential data sparseness issues – leave one country out cross-validation), these validation metrics provide a comprehensive assessment of model adequacy, calibration, and predictive accuracy.

Robustness check for potential data sparseness issues – leave one country out cross-validation As a robustness check and to address potential data sparseness issues, the final model was re-estimated using a cross-validation procedure in which each country was omitted in turn. In this process, for each cross-validation fold, the observations corresponding to the specified country were excluded from the training data, and the final model was re-estimated without these data. Model validation for each fold was then performed on both the training and test data from the regions not used in the estimation.

This cross-validation procedure, combined with the non-random splitting of the data set into training and validation sets necessitated by the spatio-temporal dependency structure, helps mitigate data sparseness problems and ensures valid estimates even in areas with potential data sparseness issues.

Supplementary Table 3: Response variables and (potentially) informative and uninformative covariates included in each predictor η_k when estimating the full model in the selection step.

Model term	Unit of measurement	Type	Description
Responses			
`Hb`	g/dL	Continuous	Hemoglobin (Hb) level at the day of the interview, scaled with μ_{Hb} and σ_{Hb} of the training data
`HAZ`	z-score	Continuous	Height-for-age z-score (HAZ) at the day of the interview defining stunting, scaled with μ_{HAZ} and σ_{HAZ} of the training data
`WAZ`	z-score	Continuous	Weight-for-age z-score (WAZ) at the day of the interview defining underweight, scaled with μ_{WAZ} and σ_{WAZ} of the training data
`WHZ`	z-score	Continuous	Weight-for-height z-score (WHZ) day of the interview defining wasting, scaled with μ_{WHZ} and σ_{WHZ} of the training data
Demographic covariates			
$\texttt{gf} \cdot \gamma_1$	-1 if "male"; 1 if "female"	Binary	Sex of the child
$\texttt{vaccine} \cdot \gamma_2$	Discretization of number of vaccinations	Categorical	Categories: "no vaccinations"; "some vaccinations" i.e, between one and five; "most vaccinations" i.e, more than five
$\texttt{education} \cdot \gamma_3$	Discretization of years of education	Categorical	Categories: "one year or less"; "between two and five years"; "six years and more"
$f_1(\texttt{ai})$	Index	Continuous	Asset index of the household
$f_2(\texttt{bord})$	Count	Metric	Birth order within household
$f_3(\texttt{cage})$	Months	Metric	Age of child
$f_4(\texttt{hhs})$	Count	Metric	Household size
$f_5(\texttt{l\_gdp})$	US$	Continuous	log(GDP) of the country
$f_6(\texttt{magebirth})$	Years	Metric	Age of mother at birth
Spatial covariates			
$\texttt{conf25} \cdot \gamma_4$	"yes" ≥ 5; "no" < 5	Binary	Indicator whether more than five conflicts have been reported in the past within a buffer of 25 km
$\texttt{conf50} \cdot \gamma_5$	"yes" ≥ 5; "no" < 5	Binary	Indicator whether more than five conflicts have been reported in the past within a buffer of 50 km,
$\texttt{conf100} \cdot \gamma_6$	"yes" ≥ 5; "no" < 5	Binary	Indicator whether more than five conflicts have been reported in the past within a buffer of 100 km
$\texttt{lc12} \cdot \gamma_7$	Land-cover classification	Categorical	Land-cover classification
$\texttt{soil2} \cdot \gamma_8$	Soil type classification	Categorical	Soil type classification
$\texttt{nl20\_12} \cdot \gamma_9$	Discretization of night-time light index	Categorical	Categories: "little light" (night-time light index $\leq$ 5 within a buffer of 20 km around the PSU); "light" (night-time light index $>$ 5 within a buffer of 20 km around the PSU)
$f_7(\texttt{altitude})$	Meters	Continuous	Altitude in m above sea level
$f_8(\texttt{l\_distance})$	Kilometers	Continuous	$log(1 +$ distance to closest body of water)
$f_9(\texttt{l\_ttcity})$	Minutes	Continuous	$log(1 +$ travel time to city)
$f_{10}(\texttt{l\_ttmotor})$	Minutes	Continuous	$log(1 +$ travel time to healthcare facility by motorized vehicle)
$f_{11}(\texttt{l\_ttwalk})$	Minutes	Continuous	$log(1 +$ travel time to healthcare facility by foot)
$f_{12}(\texttt{minc12})$	Prevalence	Continuous	Malaria incidence
$f_{13}(\texttt{ndvi12})$	Index	Continuous	Normalized difference vegetation index
$f_{14}(\texttt{pre12})$	Meters	Continuous	Precipitation
$f_{15}(\texttt{iyear})$	Years	Metric	Year of the survey interview
$f_{16}(\texttt{t2m12})$	Kelvin	Continuous	2 m surface temperature
$f_{17}(\texttt{x, y})$	Degree (lon/lat)	Continuous	Interaction longitude and latitude
$f_{18}(\texttt{x, y, cage})$	Degree (lon/lat), Metric	Continuous	Interaction longitude, latitude, and age of child
$f_{19}(\texttt{x, y, iyear})$	Degree (lon/lat) Metric	Continuous	Interaction longitude, latitude, and interview year

Note that before estimation the variables included in the response vector are scaled using their mean and standard deviation.

Supplementary Note 3 Regression diagnostic and model validation

Supplementary Note 3.1 Residual diagnostic

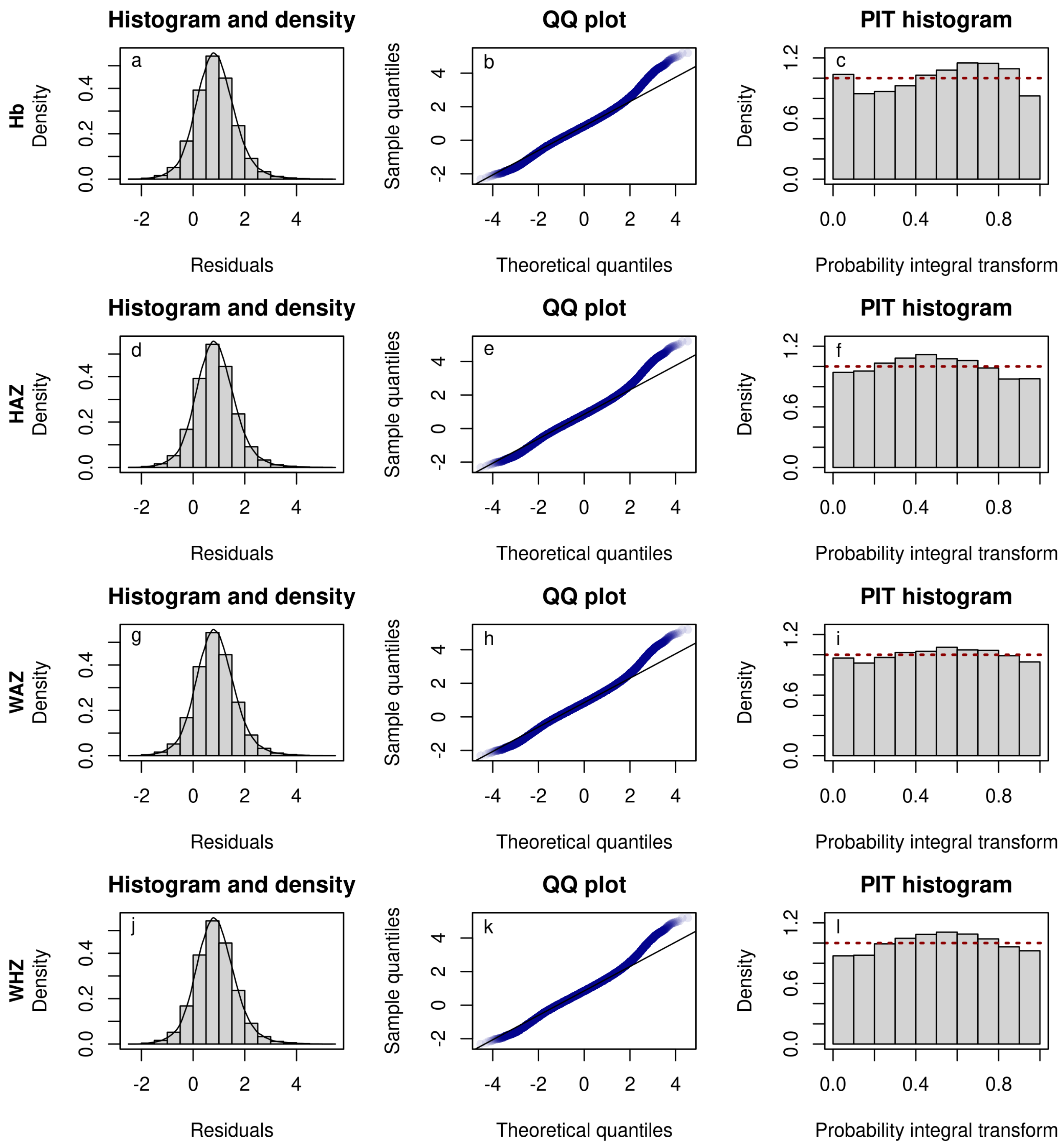

Supplementary Figure 3: **In-sample residual diagnostic based on final MCMC model.** The three columns show histograms of the marginal quantile residuals (left), QQ-plots of the marginal distributions (center), and marginal PIT histograms (right) for **a** − **c** Hb level, **d** − **f** HAZ, **g** − **i** WAZ, and; **j** − **l** WHZ. In-sample validation based on the training data (n = 164,300). For more details, see *Supplementary Figure* 1.

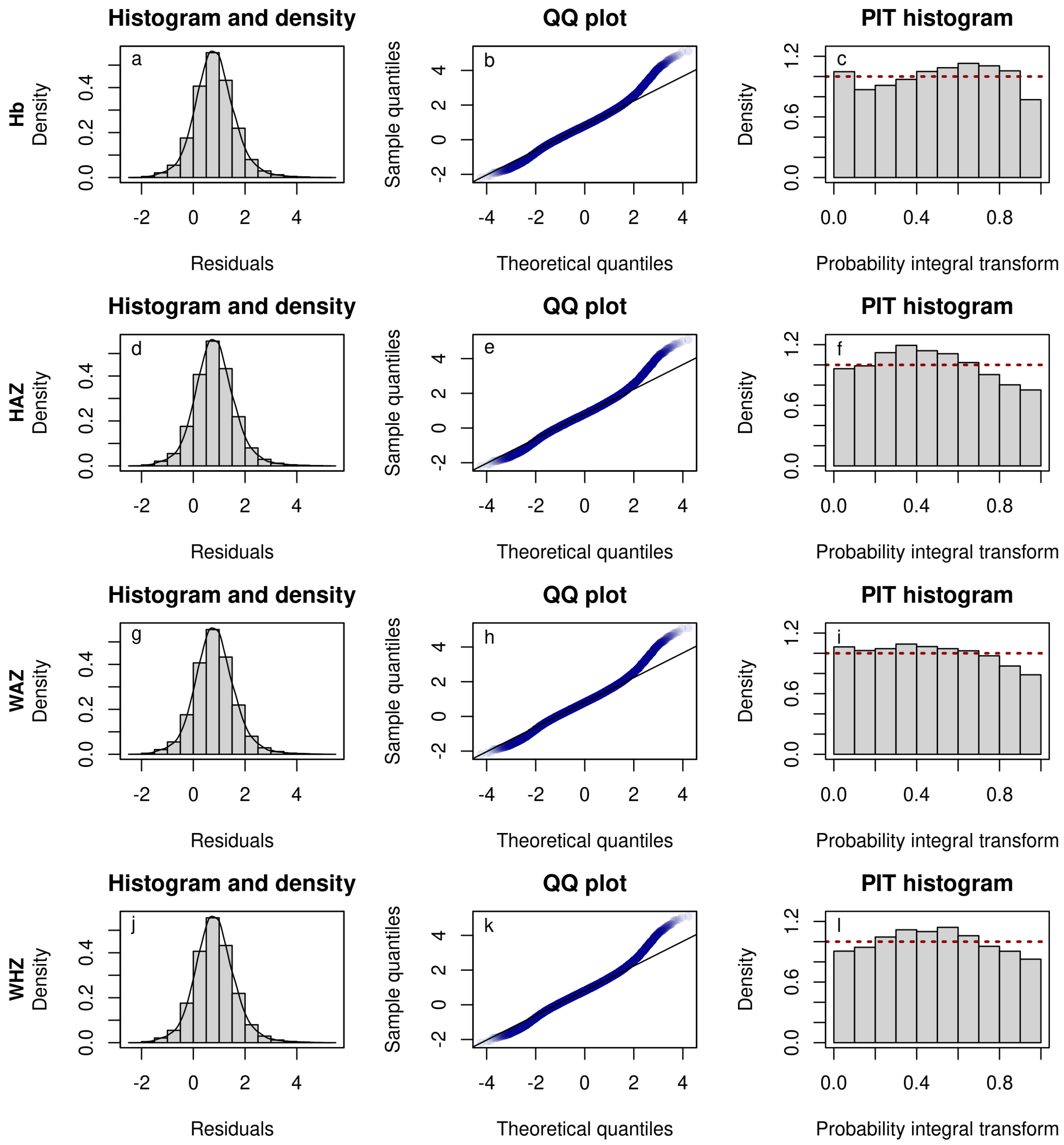

Supplementary Figure 4: **Out-of-sample residual diagnostic based on final MCMC model.** The three columns show histograms of the marginal quantile residuals (left), QQ-plots of the marginal distributions (center), and marginal PIT histograms (right) for **a** − **c** Hb level, **d** − **f** HAZ, **g** − **i** WAZ, and; **j** − **l** WHZ. Out-of-sample validation based on the test data (n = 41,074). For more details, see *Supplementary Figure* 1.

Supplementary Note 3.2 Cross-validation

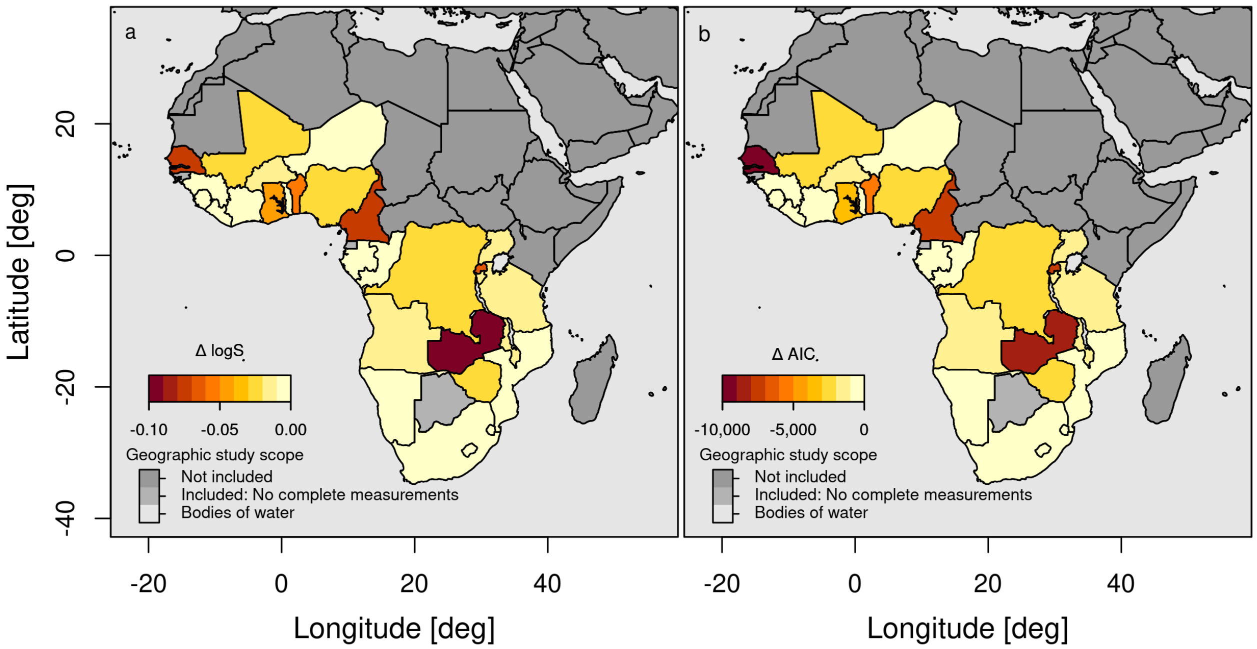

Supplementary Figure 5: **Change in validation metrics based on leave one country out cross-validation. a** change in the *logS* for all cross-validation folds against the corresponding *logS* of the final model; **b** change in the *AIC* for all cross-validation folds against the corresponding *AIC* of the final model. Note that, in both panels, the negative sign implies a relative increase and thus a worsening of the corresponding metric (calculated on the hold-out data of a specific fold) compared to the corresponding metric of the final model that has been calculated on the same hold-out data. Note that the sample size in the training and test data used in the cross-validation routine varies by fold. For more details, see *Supplementary Note* 2.

Supplementary Note 4 Software details

Software The results of this paper have been accomplished using custom software, tailored to be used at the high performance (HPC) infrastructure *LEO* of the University of Innsbruck. For that purpose the statistical software R [64] using the following R packages has been used: **bamlss** [50, 51, 55], **backports** [65], **broom** [66], **coda** [67], **codetools** [68], **colorspace** [69, 70], **deldir** [71], **dismo** [72], **gamlss.dist** [73], **maps** [74], **mgcv** [47, 75], **mvnchol** [39], **mvtnorm** [76], **nlme** [77, 78], **pillar** [79], **raster** [80], **rgeos** [81], **rgdal** [82], **rnaturalearth** [83], **rnaturalearthdata** [84], **rnaturalearthhires** [85], **scales** [86], **scoringRules** [87], **sf** [88, 89], **smoothr** [90], **sp** [91, 92]. The custom R-code used for statistical analysis is freely available in a Zenodo repository: DOI 10.5281/zenodo.18087416 [93]. This repository also includes a *README* file containing further information on how to use the code.

Computational details The custom computer code is freely available in a Zenodo repository (DOI 10.5281/zenodo.18087416 [93]). Please note that the code is custom tailored to the HPC infrastructure *LEO* of the University Innsbruck and adaptions to other systems may be required.

Supplementary Note 5 Additional results

Supplementary Note 5.1 Additional prevalence estimates

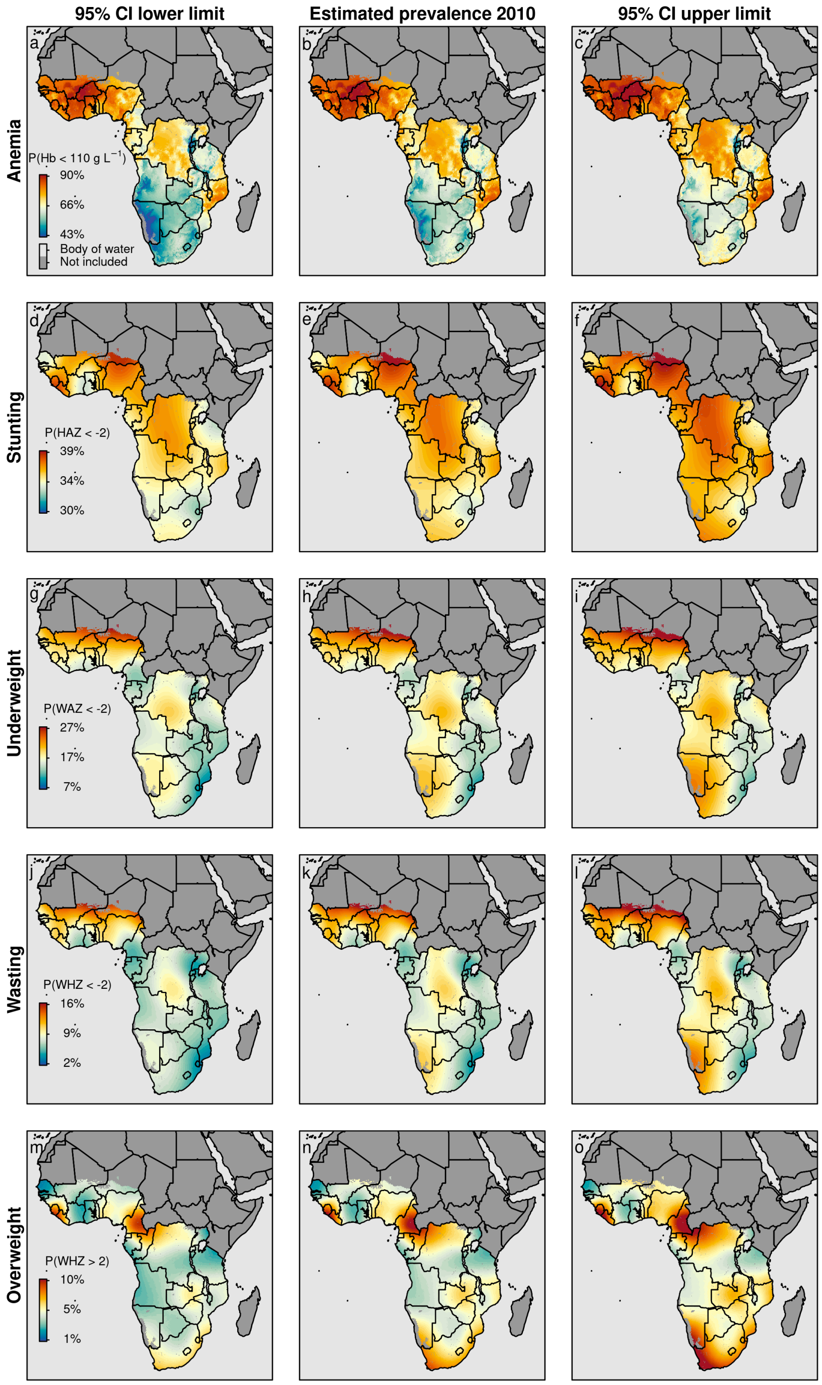

Supplementary Figure 6: **Estimated prevalence of anemia, stunting, underweight, wasting, and overweight among children aged six to 59 months in SSA in 2010.** The mean estimate (center column) is enclosed by the 95% credible intervals (left and right column). **a − c** anemia (i.e., $P(\text{Hb} < 110\, g\, L^{-1})$); **d − f** stunting (i.e., $P(\text{HAZ} < -2)$; **g − i** underweight (i.e., $P(\text{WAZ} < -2)$; **j − l** wasting (i.e., $P(\text{WHZ} < -2)$; and **m − o** overweight (i.e., $P(\text{WAZ} > 2)$. Black lines reflect country-level administrative borders. Pixels categorized as *Barren*, or *Permanent Snow and Ice*, and pixels above 3,000 m are flagged as *Not included*. Note that these predictions are based on the final model, which was fitted using the training data (n = 164,300) and validated using the test data (n = 41,074). For more details, see *Supplementary Figure* 1.

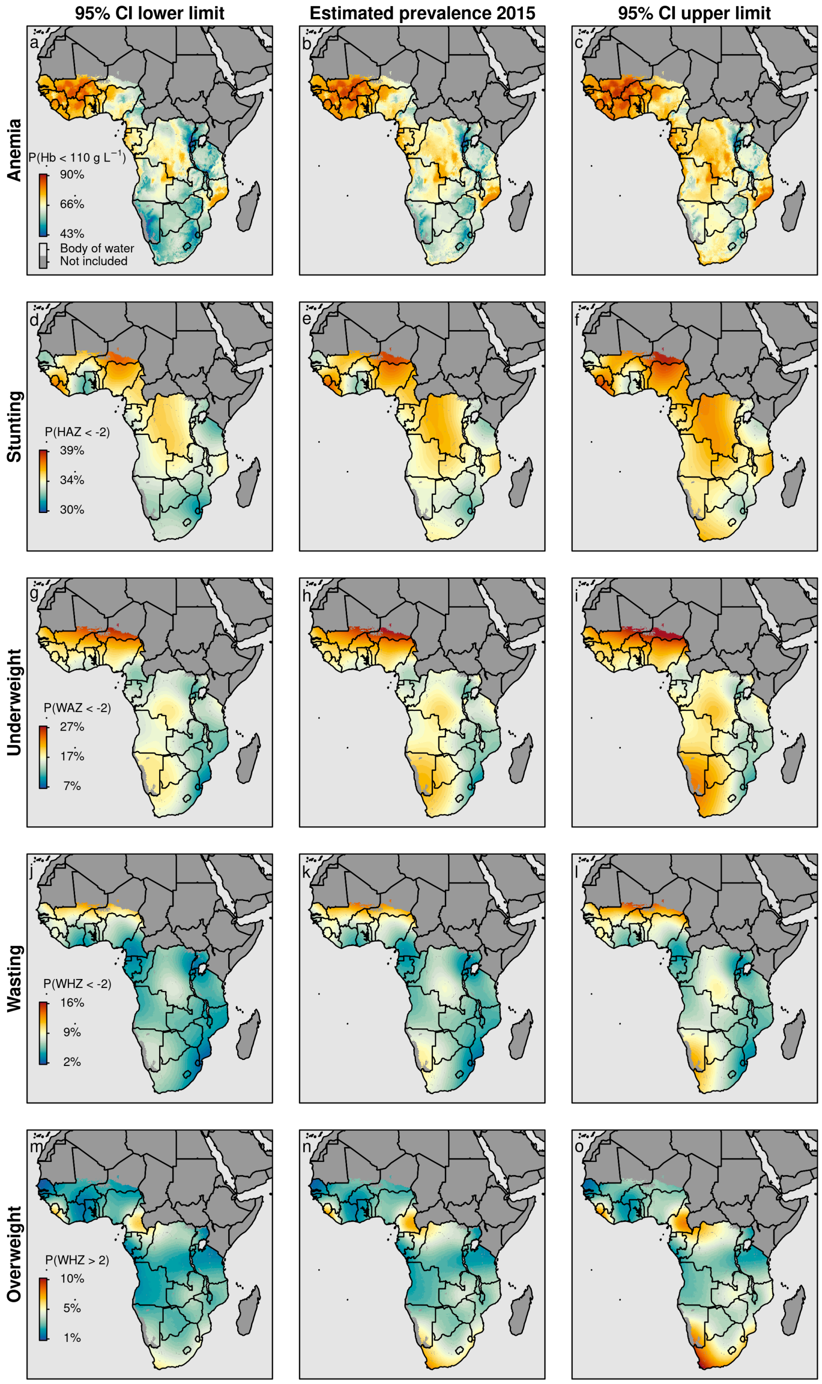

Supplementary Figure 7: Estimated prevalence of anemia, stunting, underweight, wasting, and overweight among children aged six to 59 months in SSA in 2015. The mean estimate (center column) is enclosed by the 95% credible intervals (left and right column). **a** − **c** anemia (i.e., $P(\mathrm{Hb} < 110\,g\,L^{-1})$); **d** − **f** stunting (i.e., $P(\mathrm{HAZ} < -2)$; **g** − **i** underweight (i.e., $P(\mathrm{WAZ} < -2)$; **j** − **l** wasting (i.e., $P(\mathrm{WHZ} < -2)$; and **m** − **o** overweight (i.e., $P(\mathrm{WAZ} > 2)$. Black lines reflect country-level administrative borders. Pixels categorized as *Barren*, or *Permanent Snow and Ice*, and pixels above 3,000 m are flagged as *Not included*. Note that these predictions are based on the final model, which was fitted using the training data (n = 164,300) and validated using the test data (n = 41,074). For more details, see *Supplementary Figure* 1.

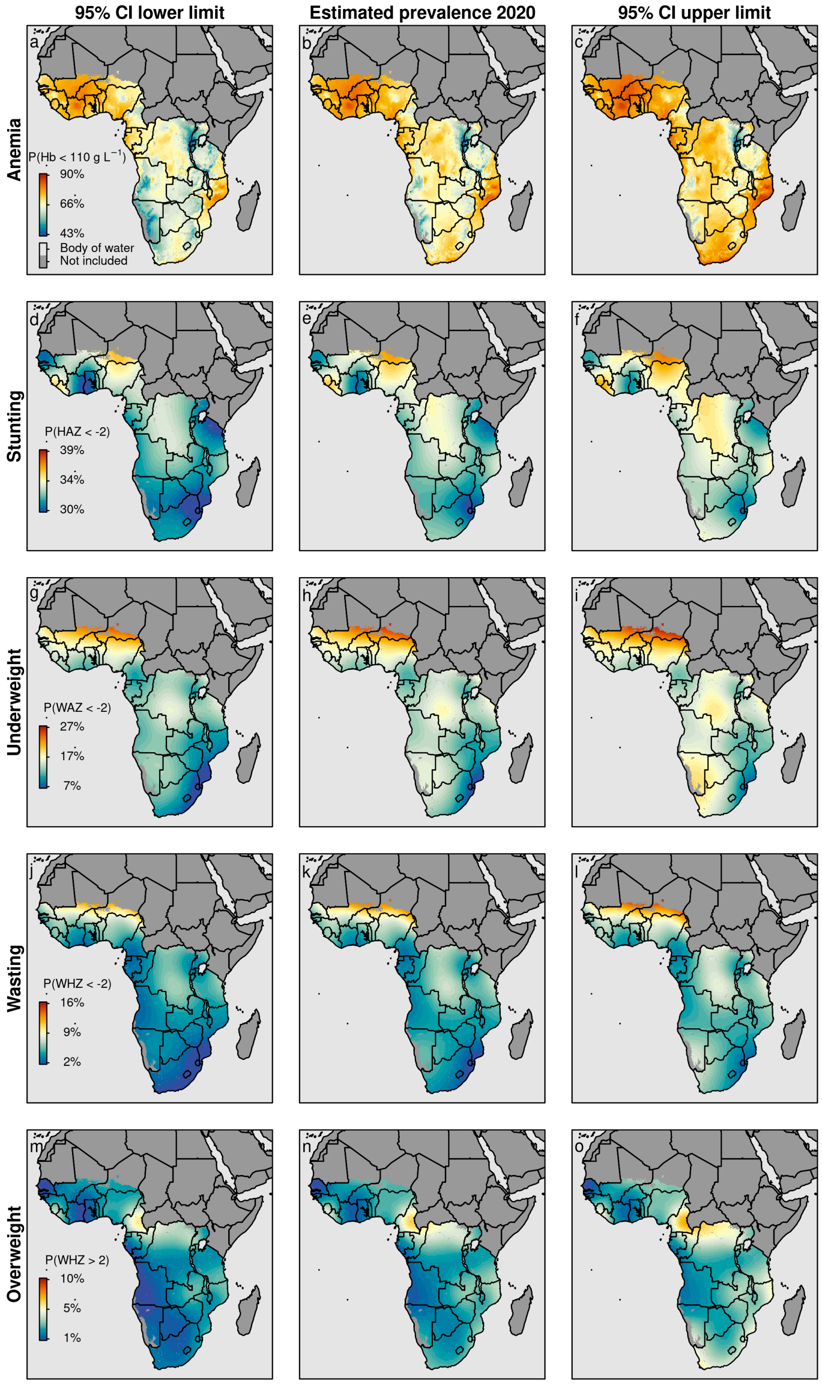

Supplementary Figure 8: **Estimated prevalence of anemia, stunting, underweight, wasting, and overweight among children aged six to 59 months in SSA in 2020.** The mean estimate (center column) is enclosed by the 95% credible intervals (left and right column). **a** − **c** anemia (i.e., $P(\text{Hb} < 110\, g\, L^{-1})$); **d** − **f** stunting (i.e., $P(\text{HAZ} < -2)$; **g** − **i** underweight (i.e., $P(\text{WAZ} < -2)$; **j** − **l** wasting (i.e., $P(\text{WHZ} < -2)$; and **m** − **o** overweight (i.e., $P(\text{WAZ} > 2)$. Black lines reflect country-level administrative borders. Pixels categorized as *Barren*, or *Permanent Snow and Ice*, and pixels above 3,000 m are flagged as *Not included*. Note that these predictions are based on the final model, which was fitted using the training data (n = 164,300) and validated using the test data (n = 41,074). For more details, see *Supplementary Figure* 1.

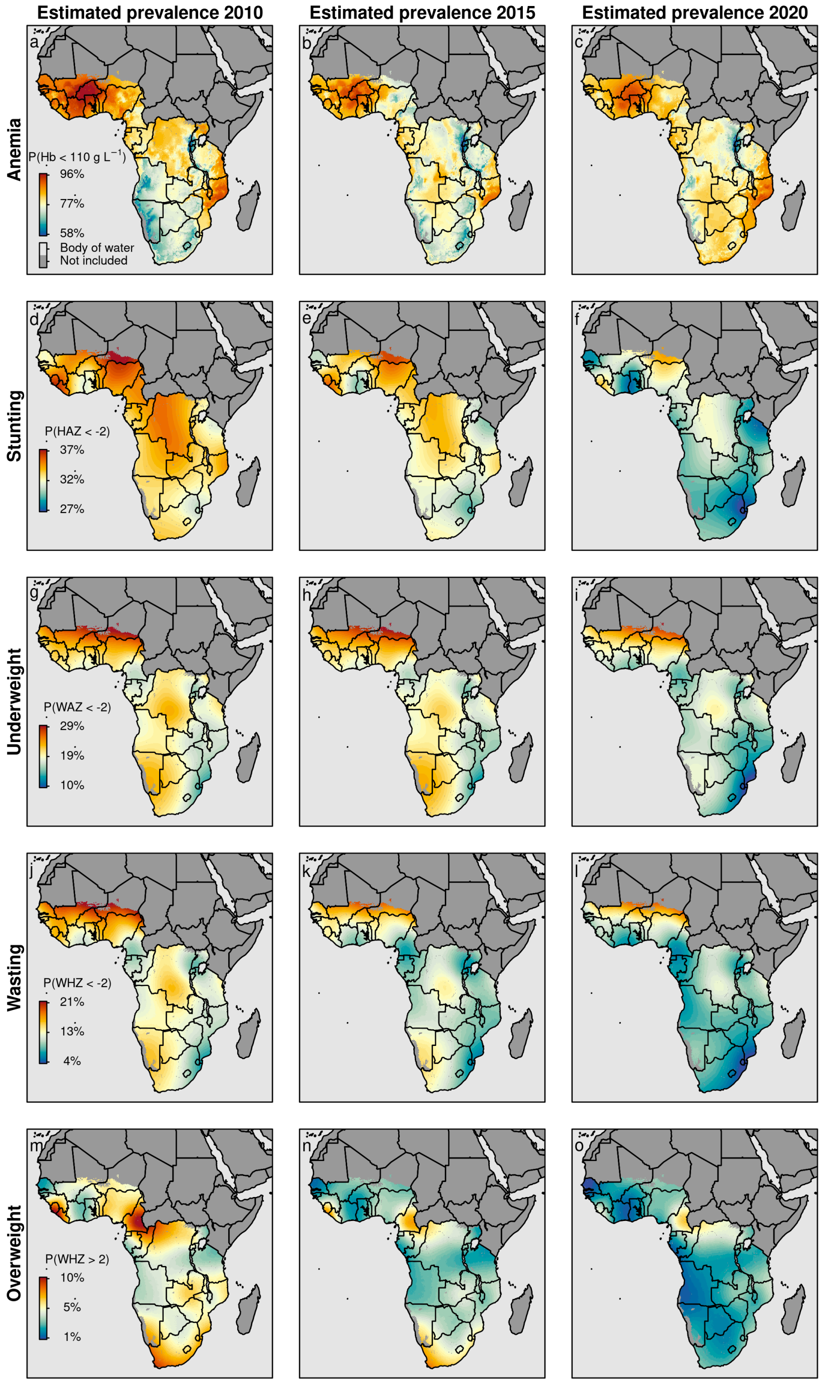

Supplementary Figure 9: **Estimated prevalence of anemia, stunting, underweight, wasting, and overweight among children aged six to 23 months in SSA in 2010, 2015, and 2020.** $\mathbf{a-c}$ anemia (i.e., $P(\text{Hb} < 110\,g\,L^{-1})$); $\mathbf{d-f}$ stunting (i.e., $P(\text{HAZ} < -2)$; $\mathbf{g-i}$ underweight (i.e., $P(\text{WAZ} < -2)$; $\mathbf{j-l}$ wasting (i.e., $P(\text{WHZ} < -2)$; and $\mathbf{m-o}$ overweight (i.e., $P(\text{WAZ} > 2)$. Black lines reflect country-level administrative borders. Pixels categorized as *Barren*, or *Permanent Snow and Ice*, and pixels above 3,000 m are flagged as *Not included*. Note that these predictions are based on the final model, which was fitted using the training data (n = 164,300) and validated using the test data (n = 41,074). For more details, see *Supplementary Figure* 1.

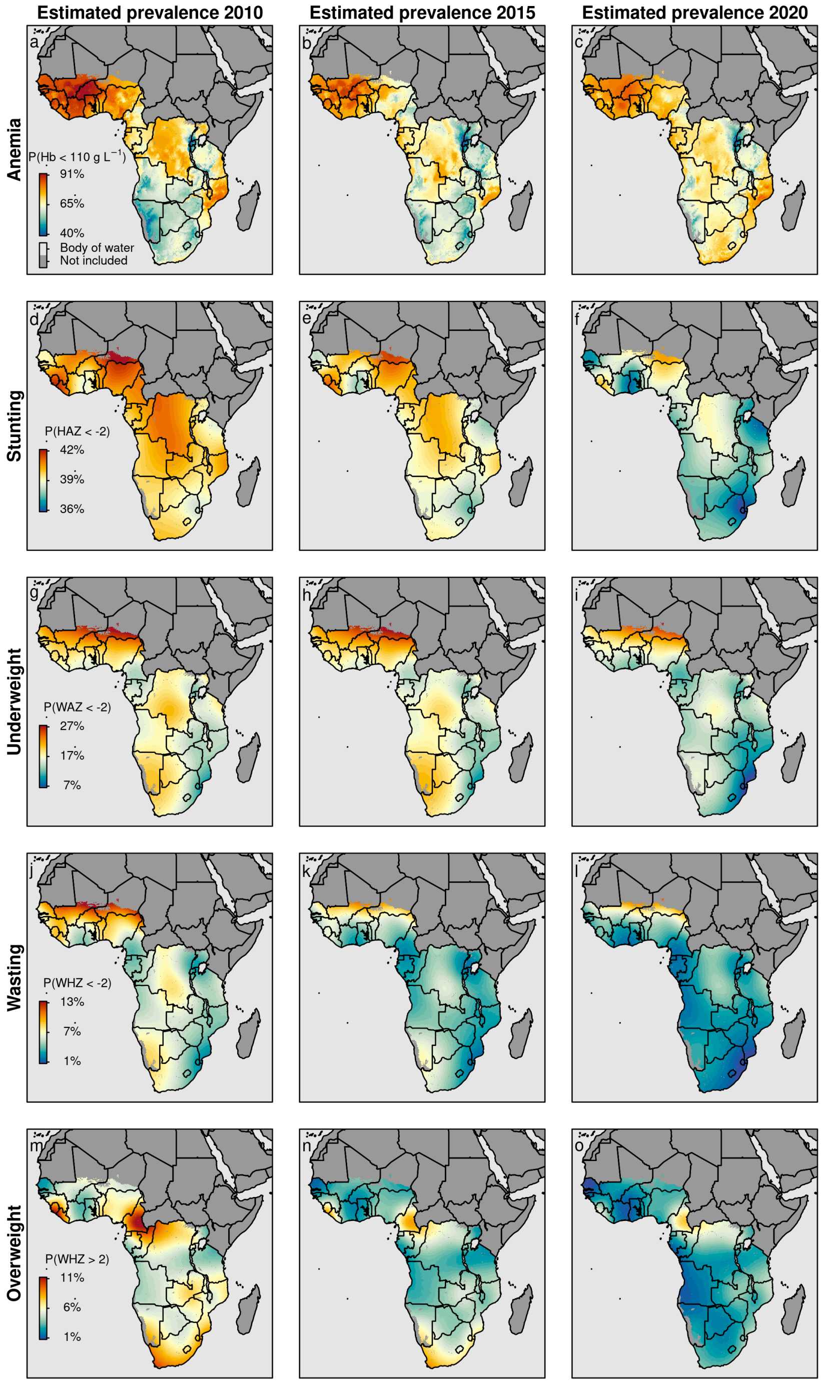

Supplementary Figure 10: **Estimated prevalence of anemia, stunting, underweight, wasting, and overweight among children aged 24 to 41 months in SSA in 2010, 2015, and 2020.** **a – c** anemia (i.e., $P(\text{Hb} < 110\, g\, L^{-1})$); **d – f** stunting (i.e., $P(\text{HAZ} < -2)$; **g – i** underweight (i.e., $P(\text{WAZ} < -2)$; **j – l** wasting (i.e., $P(\text{WHZ} < -2)$; and **m – o** overweight (i.e., $P(\text{WAZ} > 2)$. Black lines reflect country-level administrative borders. Pixels categorized as *Barren*, or *Permanent Snow and Ice*, and pixels above 3,000 m are flagged as *Not included*. Note that these predictions are based on the final model, which was fitted using the training data (n = 164,300) and validated using the test data (n = 41,074). For more details, see *Supplementary Figure* 1.

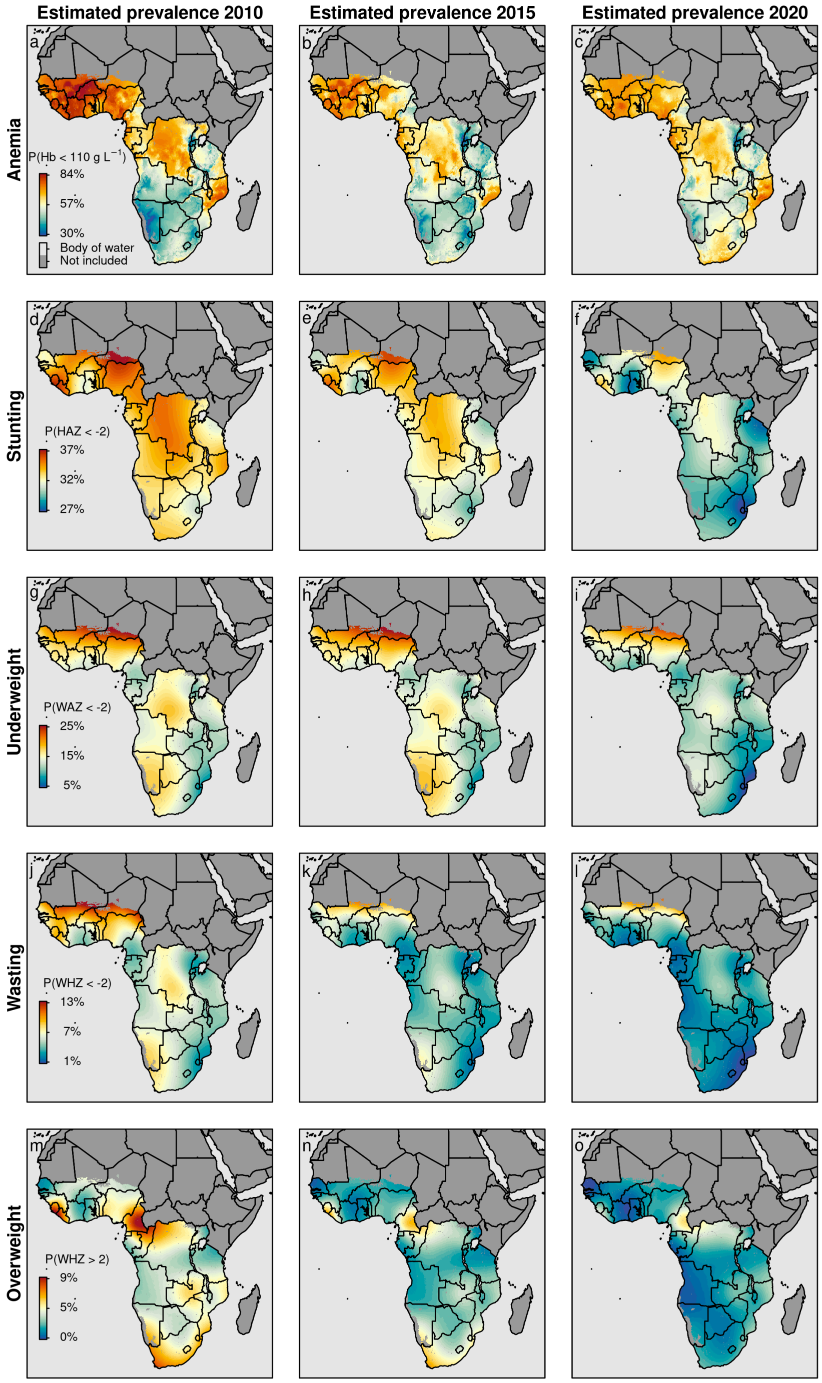

Supplementary Figure 11: **Estimated prevalence of anemia, stunting, underweight, wasting, and overweight among children aged 42 to 59 months in SSA in 2010, 2015, and 2020.** $\mathbf{a-c}$ anemia (i.e., $P(\text{Hb} < 110\, g\, L^{-1})$); $\mathbf{d-f}$ stunting (i.e., $P(\text{HAZ} < -2)$; $\mathbf{g-i}$ underweight (i.e., $P(\text{WAZ} < -2)$; $\mathbf{j-l}$ wasting (i.e., $P(\text{WHZ} < -2)$; and $\mathbf{m-o}$ overweight (i.e., $P(\text{WAZ} > 2)$. Black lines reflect country-level administrative borders. Pixels categorized as *Barren*, or *Permanent Snow and Ice*, and pixels above 3,000 m are flagged as *Not included*. Note that these predictions are based on the final model, which was fitted using the training data (n = 164,300) and validated using the test data (n = 41,074). For more details, see *Supplementary Figure* 1.

Supplementary Note 5.2 Additional estimates pairwise correlation

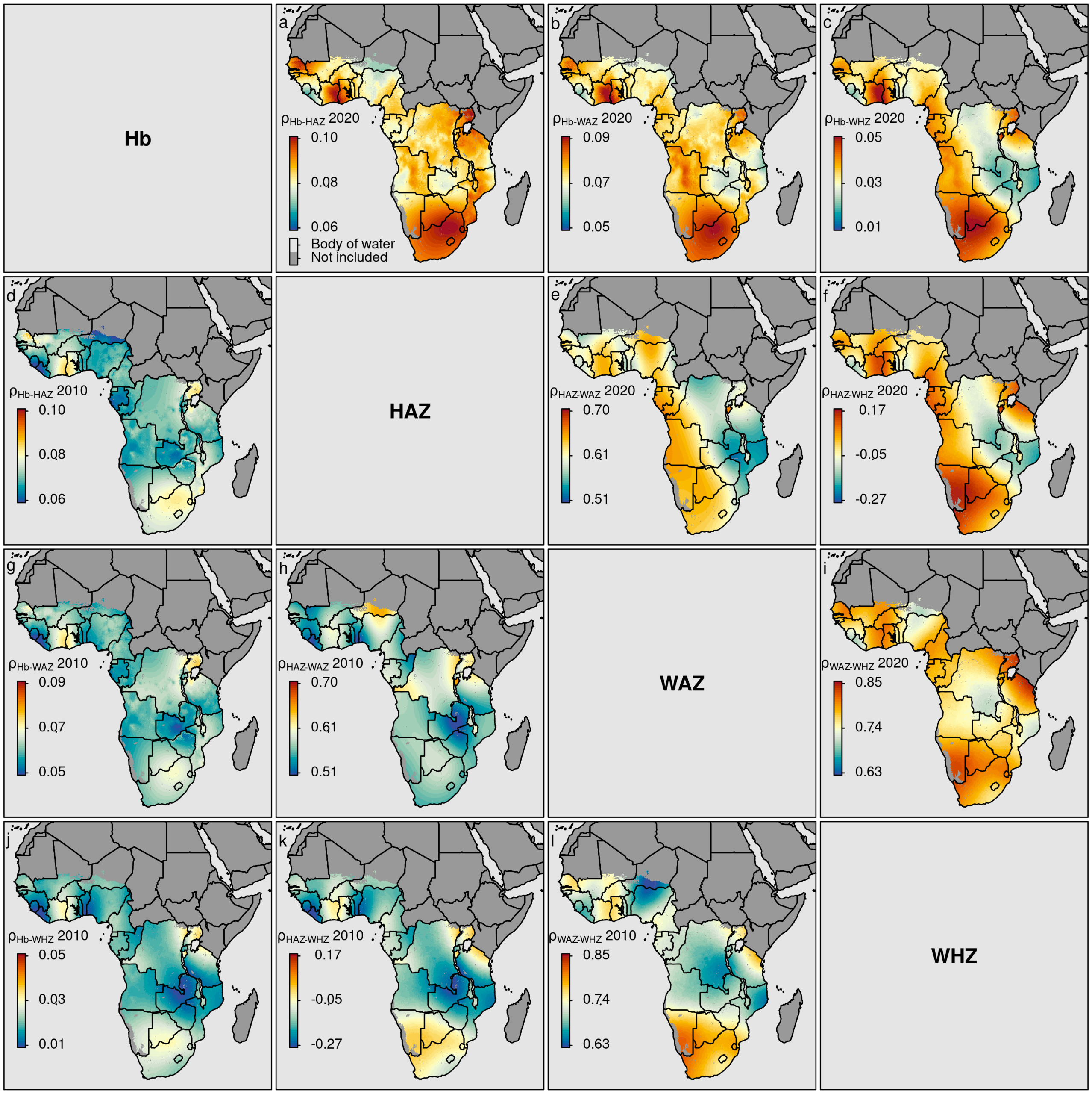

Supplementary Figure 12: **Estimated pairwise correlation between four key nutritional indicators among children aged six to 23 months in SSA. a** Hb level and HAZ, 2020; **b** Hb level and WAZ, 2020; **c** Hb level and WHZ, 2020; **d** Hb level and HAZ, 2010; **e** HAZ and WAZ, 2020; **f** HAZ and WHZ, 2020; **g** Hb level and WAZ, 2010; **h** HAZ and WAZ, 2010; **i** WAZ and WHZ, 2020; **j** Hb level and WHZ, 2010; **k** HAZ and WHZ, 2010; **l** WAZ and WHZ, 2010; Black lines reflect country-level administrative borders. Pixels categorized as *Barren*, or *Permanent Snow and Ice*, and pixels above 3,000 m are flagged as *Not included*. Note that these predictions are based on the final model, which was fitted using the training data (n = 164,300) and validated using the test data (n = 41,074). For more details, see *Supplementary Figure* 1.

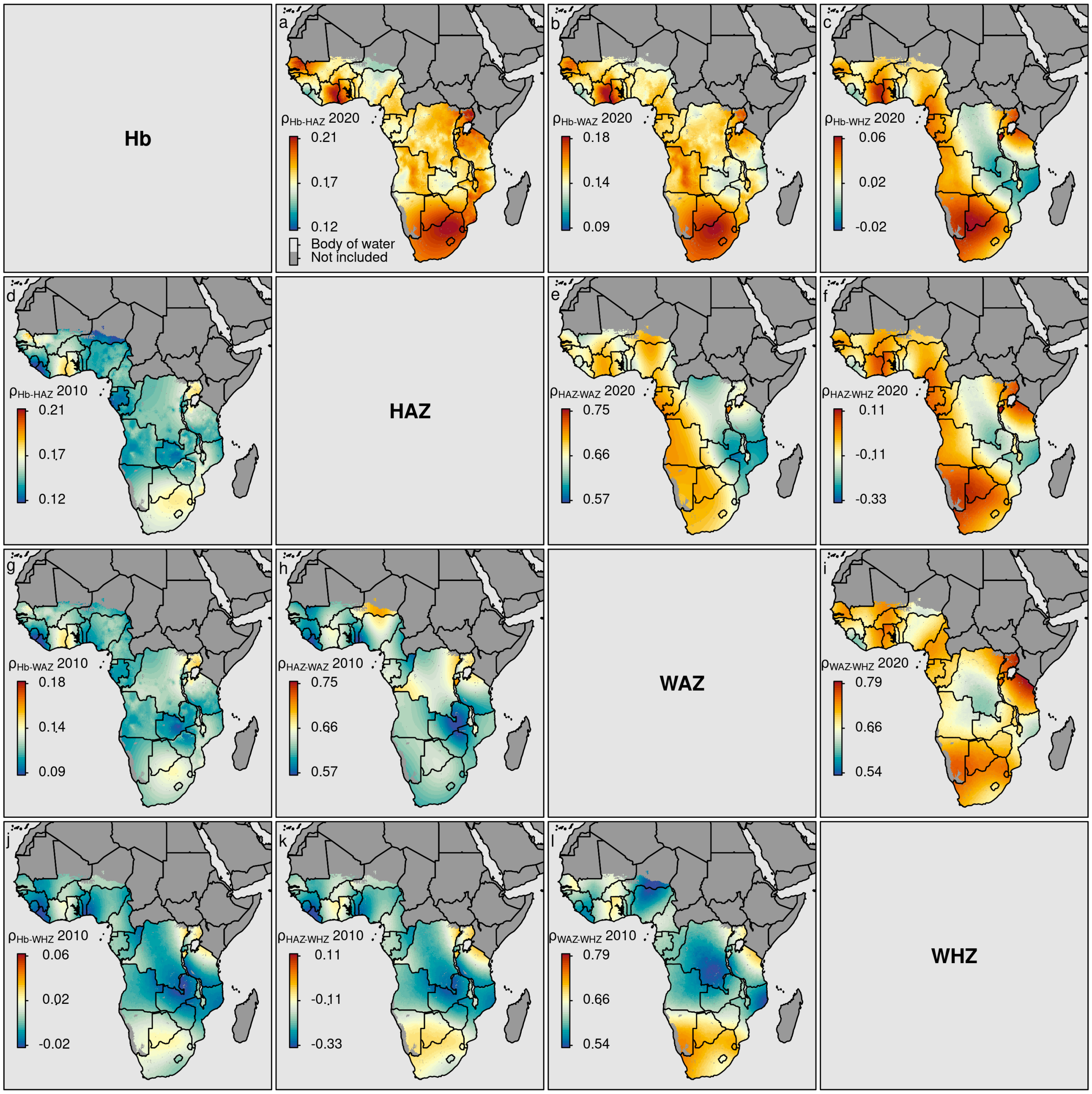

Supplementary Figure 13: **Estimated pairwise correlation between four key nutritional indicators among children aged 24 to 41 months in SSA. a** Hb level and HAZ, 2020; **b** Hb level and WAZ, 2020; **c** Hb level and WHZ, 2020; **d** Hb level and HAZ, 2010; **e** HAZ and WAZ, 2020; **f** HAZ and WHZ, 2020; **g** Hb level and WAZ, 2010; **h** HAZ and WAZ, 2010; **i** WAZ and WHZ, 2020; **j** Hb level and WHZ, 2010; **k** HAZ and WHZ, 2010; **l** WAZ and WHZ, 2010; Black lines reflect country-level administrative borders. Pixels categorized as *Barren*, or *Permanent Snow and Ice*, and pixels above 3,000 m are flagged as *Not included*. Note that these predictions are based on the final model, which was fitted using the training data (n = 164,300) and validated using the test data (n = 41,074). For more details, see *Supplementary Figure* 1.

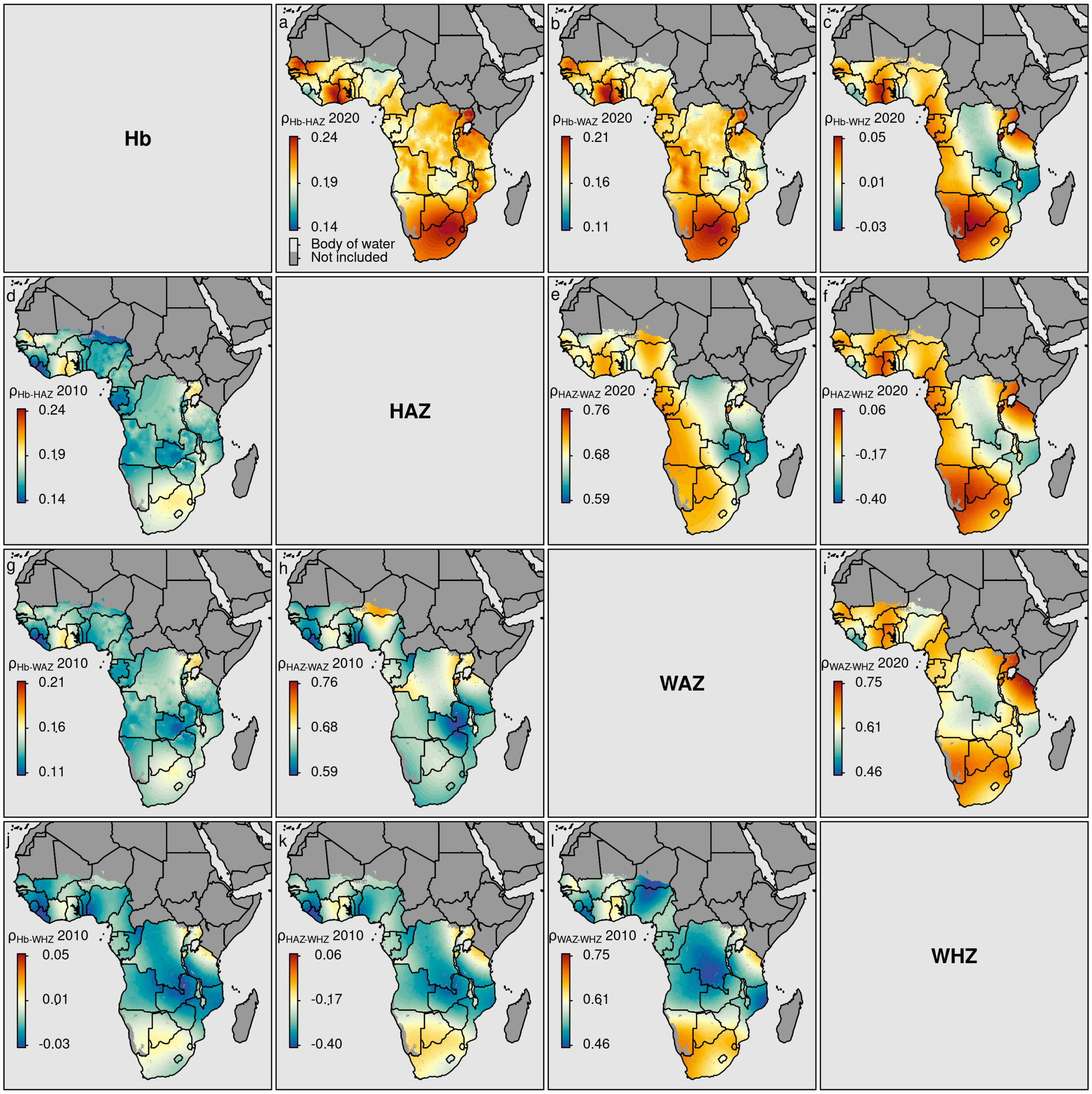

Supplementary Figure 14: **Estimated pairwise correlation between four key nutritional indicators among children aged 42 to 59 months in SSA. a** Hb level and HAZ, 2020; **b** Hb level and WAZ, 2020; **c** Hb level and WHZ, 2020; **d** Hb level and HAZ, 2010; **e** HAZ and WAZ, 2020; **f** HAZ and WHZ, 2020; **g** Hb level and WAZ, 2010; **h** HAZ and WAZ, 2010; **i** WAZ and WHZ, 2020; **j** Hb level and WHZ, 2010; **k** HAZ and WHZ, 2010; **l** WAZ and WHZ, 2010; Black lines reflect country-level administrative borders. Pixels categorized as *Barren*, or *Permanent Snow and Ice*, and pixels above 3,000 m are flagged as *Not included*. Note that these predictions are based on the final model, which was fitted using the training data (n = 164,300) and validated using the test data (n = 41,074). For more details, see *Supplementary Figure* 1.

Supplementary References

[1] Amante, C. & Eakins, B. ETOPO1 1 arc-minute global relief model: Procedures, data sources and analysis (2009). Retrieved January 27, 2021, from NOAA Technical Memorandum NESDIS NGDC-24 doi:10.7289/V5C8276M.

[2] NOAA National Geophysical Data Center. ETOPO1 1 arc-minute global relief model [Dataset] (2009). Retrieved January 27, 2021, from https://www.ncei.noaa.gov/access/metadata/landing-page/bin/iso?id=gov.noaa.ngdc.mgg.dem:316.

[3] Davies, S., Pettersson, T. & Öberg, M. Organized violence 1989–2021 and drone warfare. *Journal of Peace Research* **59**, 593–610 (2022). doi:10.1177/00223433221108428.

[4] Sundberg, R. & Melander, E. Introducing the UCDP georeferenced event dataset. *Journal of Peace Research* **50**, 523–532 (2013). doi:10.1177/0022343313484347.

[5] The World Bank. World Development Indicators 2022 (2022). Retrieved November 04, 2022, from https://data.worldbank.org/.

[6] Friedl, M. A. *et al.* MODIS collection 5 global land cover: Algorithm refinements and characterization of new datasets. *Remote Sensing of Environment* **114**, 168–182 (2010). doi:10.1016/j.rse.2009.08.016.

[7] Friedl, M. & Sulla-Menashe, D. MCD12C1 MODIS/Terra+Aqua land cover type yearly L3 global 0.05Deg CMG V006 [Dataset] (2015). Retrieved July 07, 2022, from doi:10.5067/MODIS/MCD12C1.006.

[8] Weiss, D. J. *et al.* Mapping the global prevalence, incidence, and mortality of *Plasmodium falciparum*, 2000-17: A spatial and temporal modelling study. *Lancet* **394**, 322–331 (2019). doi:10.1016/S0140-6736(19)31097-9.

[9] Battle, K. *et al.* Mapping the global endemicity and clinical burden of *Plasmodium vivax*, 2000-17: A spatial and temporal modelling study. *Lancet* **394**, 332–343 (2019). doi:10.1016/S0140-6736(19)31096-7.

[10] Gething, P. W. *et al.* Modelling the global constraints of temperature on transmission of *Plasmodium falciparum* and *P. vivax*. *Parasites & Vectors* **4**, 92 (2011). doi:10.1186/1756-3305-4-92.

[11] Gething, P. W. *et al.* A new world malaria map: *Plasmodium falciparum* endemicity in 2010. *Malaria Journal* **10**, 378 (2011). doi:10.1186/1475-2875-10-378.

[12] Gething, P. W. *et al.* A long neglected world malaria map: *Plasmodium vivax* endemicity in 2010. *PLOS Neglected Tropical Diseases* **6**, e1814 (2012). doi:10.1371/journal.pntd.0001814.

[13] Li, X., Zhou, Y., Zhao, M. & Zhao, X. A harmonized global nighttime light dataset 1992-2018. *Scientific Data* **7**, 168 (2020). doi:10.1038/s41597-020-0510-y.

[14] Tucker, C. J. *et al.* An extended AVHRR 8-km NDVI dataset compatible with MODIS and SPOT vegetation NDVI data. *International Journal of Remote Sensing* **26**, 4485–4498 (2005). doi:10.1080/01431160500168686.

[15] Pinzon, J. E. & Tucker, C. J. A non-stationary 1981–2012 AVHRR $NDVI_{3g}$ time series. *Remote Sensing* **6**, 6929–6960 (2014). doi:10.3390/rs6086929.

[16] Didan, K. & Barreto, A. Nasa measures vegetation index and phenology (VIP) phenology EVI2 yearly global 0.05Deg CMG [Dataset] (2016). doi:10.5067/MEaSUREs/VIP/VIPPHEN_EVI2.004.

[17] Hersbach, H. *et al.* The ERA5 global reanalysis. *Quarterly Journal of the Royal Meteorological Society* **146**, 1999–2049 (2020). doi:10.1002/qj.3803.

[18] Poggio, L. *et al.* SoilGrids 2.0: Producing soil information for the globe with quantified spatial uncertainty [Dataset]. *SOIL* **7**, 217–240 (2020). doi:10.5194/soil-7-217-2021.

[19] Weiss, D. J. *et al.* Global maps of travel time to healthcare facilities. *Nature Medicine* **26**, 1835–1838 (2020). doi:10.1038/s41591-020-1059-1.

[20] Weiss, D. J. *et al.* A global map of travel time to cities to assess inequalities in accessibility in 2015. *Nature* **553**, 333–336 (2018). doi:10.1038/nature25181.

[21] NASA JPL. NASA shuttle radar topography mission water body data shapefiles & raster files [Dataset] (2013). Retrieved May 25, 2021, from doi:10.5067/MEaSUREs/SRTM/SRTMSWBD.003.

[22] ICF. Demographic and Health Surveys (various) [Datasets] (2004-2017). Funded by USAID. Accessible via `https://dhsprogram.com/Data/`.

[23] DHS. Description of the Demographic and Health Surveys Program: The DHS Program (2019). Retrieved September 26, 2019, from `https://dhsprogram.com/Data/Guide-to-DHS-Statistics/Description_of_The_Demographic_and_Health_Surveys_Program.htm`.

[24] World Health Organization (WHO). Global malaria programme (2021). Retrieved May 24, 2024, from `https://www.who.int/teams/global-malaria-programme/elimination/countries-and-territories-certified-malaria-free-by-who`.

[25] Hastie, T. J. & Tibshirani, R. J. *Generalized Additive Models* (Chapman & Hall/CRC, Boca Raton, London, New York, Washington D.C., 1990), 1st edn. doi:10.1201/9780203753781.

[26] Rigby, R. A. & Stasinopoulos, D. M. Generalized additive models for location, scale and shape. *Journal of the Royal Statistical Society: Series C (Applied Statistics)* **54**, 507–554 (2005). doi:10.1111/j.1467-9876.2005.00510.x.

[27] Klein, N., Kneib, T. & Lang, S. Bayesian generalized additive models for location, scale, and shape for zero-inflated and overdispersed count data. *Journal of the American Statistical Association* **110**, 405–419 (2015). doi:10.1080/01621459.2014.912955.

[28] Klein, N., Kneib, T., Lang, S. & Sohn, A. Bayesian structured additive distributional regression with an application to regional income inequality in Germany. *Annals of Applied Statistics* **9**, 1024–1052 (2015). doi:10.1214/15-AOAS823.

[29] Klein, N., Kneib, T., Klasen, S. & Lang, S. Bayesian structured additive distributional regression for multivariate responses. *Journal of the Royal Statistical Society: Series C (Applied Statistics)* **64**, 569–591 (2015). doi:10.1111/rssc.12090.

[30] Verbesselt, J. *et al.* Remotely sensed resilience of tropical forests. *Nature Climate Change* **6**, 1028–1031 (2016). doi:10.1038/nclimate3108.

[31] Stauffer, R., Mary, G. J., Messner, J. W., Umlauf, N. & Zeileis, A. Spatio-temporal precipitation climatology over complex terrain using a censored additive regression model. *International Journal of Climatology* **37**, 3264–3275 (2017). doi:10.1002/joc.4913.

[32] Adebayo, S. B. & Fahrmeir, L. Analysing child mortality in Nigeria with geoadditive discrete-time survival models. *Statistics in Medicine* **24**, 709–728 (2005). doi:10.1002/sim.1842.

[33] Golding, N. *et al.* Mapping under-5 and neonatal mortality in Africa, 2000-15: A baseline analysis for the Sustainable Development Goals. *Lancet* **390**, 2171–2182 (2017). doi:10.1016/S0140-6736(17)31758-0.

[34] Harttgen, K., Lang, S., Santer, J. & Seiler, J. Modelling under-five mortality through multilevel structured additive regression with varying coefficients for Asia and sub-Saharan Africa. *Journal of Development Studies* **56**, 401–430 (2020). doi:10.1080/00220388.2018.1563681.

[35] Kandala, N.-B., Fahrmeir, L., Klasen, S. & Priebe, J. Geo-additive models of childhood undernutrition in three sub-Saharan African countries. *Population, Space and Place* **15**, 461–473 (2009). doi:10.1002/psp.524.

[36] Osgood-Zimmerman, A. *et al.* Mapping child growth failure in Africa between 2000 and 2015. *Nature* **555**, 41–47 (2018). doi:10.1038/nature25760.

[37] Seiler, J., Harttgen, K., Kneib, T. & Lang, S. Modelling children's anthropometric status using Bayesian distributional regression merging socio-economic and remote sensed data from South Asia and sub-Saharan Africa. *Economics & Human Biology* **40**, 100950 (2021). doi:10.1016/j.ehb.2020.100950.

[38] Seiler, J., Wetscher, M., Harttgen, K., Utzinger, J. & Umlauf, N. High-resolution spatial prediction of anemia risk among children aged 6 to 59 months in low- and middle-income countries. *Communications Medicine* **5**, 57 (2025). doi:10.1038/s43856-025-00765-2.

[39] Muschinski, T., Mayr, G. J., Simon, T., Umlauf, N. & Zeileis, A. Cholesky-based multivariate Gaussian regression. *Econometrics and Statistics* **29**, 261–281 (2024). doi:10.1016/j.ecosta.2022.03.001.

[40] Kock, L. & Klein, N. Truly multivariate structured additive distributional regression. *Journal of Computational and Graphical Statistics* **00**, 1–13 (2025). doi:10.1080/10618600.2024.2434181.

[41] Umlauf, N. & Kneib, T. A primer on Bayesian distributional regression. *Statistical Modelling* **18**, 219–247 (2018). doi:10.1177/1471082X18759140.

[42] Roberts, D. R. *et al.* Cross-validation strategies for data with temporal, spatial, hierarchical, or phylogenetic structure. *Ecography* **40**, 913–929 (2017). doi:10.1111/ecog.02881.

[43] Umlauf, N. *et al.* Scalable estimation for structured additive distributional regression. *Journal of Computational and Graphical Statistics* **34**, 601–617 (2025). doi:10.1080/10618600.2024.2388604.

[44] Sakamoto, Y., Ishiguro, M. & Kitagawa, G. *Akaike Information Criterion Statistics*. Mathematics and its Applications (Springer, Dordrecht, Netherlands, 1986), 1st edn.

[45] Good, I. J. Rational decisions. *Journal of the Royal Statistical Society: Series B (Methodological)* **14**, 107–114 (1952). doi:10.1111/j.2517-6161.1952.tb00104.x.

[46] Fahrmeir, L., Kneib, T., Lang, S. & Brian, M. *Regression: Models, Methods and Applications* (Springer, Berlin, Heidelberg, 2021), 2nd edn. doi:10.1007/978-3-662-63882-8.

[47] Wood, S. N. Thin plate regression splines. *Journal of the Royal Statistical Society: Series B (Statistical Methodology)* **65**, 95–114 (2003). doi:10.1111/1467-9868.00374.

[48] Wood, S. N., Li, Z., Shaddick, G. & Augustin, N. H. Generalized additive models for gigadata: Modelling the UK black smoke network daily data. *Journal of the American Statistical Association* **112**, 1199–1210 (2017). doi:10.1080/01621459.2016.1195744.

[49] Pourahmadi, M. Joint mean-covariance models with applications to longitudinal data: unconstrained parameterisation. *Biometrika* **86**, 677–690 (1999). doi:10.1093/biomet/86.3.677.

[50] Umlauf, N., Klein, N. & Zeileis, A. BAMLSS: Bayesian additive models for location, scale, and shape (and beyond). *Journal of Computational and Graphical Statistics* **27**, 612–627 (2018). doi:10.1080/10618600.2017.1407325.

[51] Umlauf, N., Klein, N., Simon, T. & Zeileis, A. **bamlss**: A Lego toolbox for flexible Bayesian regression (and beyond). *Journal of Statistical Software* **100**, 1–53 (2021). doi:10.18637/jss.v100.i04.

[52] Gamerman, D. Sampling from the posterior distribution in generalized linear mixed models. *Statistics and Computing* **7**, 57–68 (1997). doi:10.1023/A:1018509429360.

[53] Lang, S., Umlauf, N., Wechselberger, P., Harttgen, K. & Kneib, T. Multilevel structured additive regression. *Statistics and Computing* **24**, 223–238 (2014). doi:10.1007/s11222-012-9366-0.

[54] Köhler, M., Umlauf, N. & Greven, S. Nonlinear association structures in flexible Bayesian additive joint models. *Statistics in Medicine* **37**, 4771–4788 (2018). doi:10.1002/sim.7967.

[55] Umlauf, N. *et al.* **bamlss**: Bayesian additive models for location scale and shape (and beyond) (2024). R package version 1.2-3 `http://CRAN.R-project.org/package=bamlss`.

[56] Dunn, P. K. & Smyth, G. K. Randomized quantile residuals. *Journal of Computational and Graphical Statistics* **5**, 236–244 (1996). doi:10.1080/10618600.1996.10474708.

[57] Dawid, A. P. Present position and potential developments: Some personal views statistical theory the prequential approach. *Journal of the Royal Statistical Society: Series A (General)* **147**, 278–290 (1984). doi:10.2307/2981683.

[58] Gneiting, T. & Raftery, A. E. Strictly proper scoring rules, prediction, and estimation. *Journal of the American Statistical Association* **102**, 359–378 (2007). doi:10.1198/016214506000001437.

[59] Klein, N., Kneib, T., Marra, G. & Radice, R. Bayesian mixed binary-continuous copula regression with an application to childhood undernutrition. In Fan, Y., Nott, D., Smith, M. S. & Dortet-Bernadet, J.-L. (eds.) *Flexible Bayesian Regression Modelling*, 121–152 (Academic Press, 2020). doi:10.1016/B978-0-12-815862-3.00011-1.

[60] Ziegel, J. F. & Gneiting, T. Copula calibration. *Electronic Journal of Statistics* **8**, 2619 – 2638 (2014). doi:10.1214/14-EJS964.

[61] Thorarinsdottir, T. L., Scheuerer, M. & and, C. H. Assessing the calibration of high-dimensional ensemble forecasts using rank histograms. *Journal of Computational and Graphical Statistics* **25**, 105–122 (2016). doi:10.1080/10618600.2014.977447.

[62] Pic, R., Dombry, C., Naveau, P. & Taillardat, M. Proper scoring rules for multivariate probabilistic forecasts based on aggregation and transformation. *Advances in Statistical Climatology, Meteorology and Oceanography* **11**, 23–58 (2025). doi:10.5194/ascmo-11-23-2025.

[63] Leutbecher, M. & Baran, S. Ensemble size dependence of the logarithmic score for forecasts issued as multivariate normal distributions. *Quarterly Journal of the Royal Meteorological Society* **151**, e4898 (2025). doi:10.1002/qj.4898.

[64] R Core Team. R: A language and environment for statistical computing (2023). `https://www.R-project.org/`.

[65] Lang, M. & R Core Team. **backports**: Reimplementations of functions introduced since R-3.0.0 (2020). R package version 1.4.1; `https://CRAN.R-project.org/package=backports`.

[66] Robinson, D., Hayes, A. & Couch, S. **broom**: Convert statistical objects into tidy tibbles (2021). R package version 1.0.5; `https://CRAN.R-project.org/package=broom`.

[67] Plummer, M., Best, N., Cowles, K. & Vines, K. **CODA**: Convergence diagnosis and output analysis for MCMC. *R News* **6**, 7–11 (2006). `https://journal.r-project.org/archive/`, `https://www.r-project.org/doc/Rnews/Rnews_2006-1.pdf`.

[68] Tierney, L. **codetools**: Code analysis tools for R (2020). R package version 0.2-20; `https://CRAN.R-project.org/package=codetools`.

[69] Zeileis, A., Hornik, K. & Murrell, P. Escaping RGBland: Selecting colors for statistical graphics. *Computational Statistics & Data Analysis* **53**, 3259–3270 (2009). doi:10.1016/j.csda.2008.11.033.

[70] Zeileis, A. *et al.* **colorspace**: A toolbox for manipulating and assessing colors and palettes. *Journal of Statistical Software* **96**, 1–49 (2020). doi:10.18637/jss.v096.i01.

[71] Turner, R. **deldir**: Delaunay triangulation and Dirichlet (Voronoi) tessellation (2021). R package version 2.0-4; https://CRAN.R-project.org/package=deldir.

[72] Hijmans, R. J., Phillips, S., Leathwick, J. & Elith, J. **dismo**: Species distribution modeling (2020). R package version 1.3-14; https://CRAN.R-project.org/package=dismo.

[73] Stasinopoulos, M. & Rigby, R. **gamlss.dist**: Distributions for generalized additive models for location scale and shape (2021). R package version 6.1-1; https://CRAN.R-project.org/package=gamlss.dist.

[74] Original S code by Richard A. Becker and Allan R. Wilks. R version by Ray Brownrigg. Enhancements by Thomas P. Minka and Alex Deckmyn. **maps**: Draw geographical maps (2018). R package version 3.4.2.1; https://CRAN.R-project.org/package=maps.

[75] Wood, S. N. *Generalized Additive Models: An Introduction with R* (Chapman & Hall/CRC, New York, USA, 2017), 2nd edn. doi:10.1201/9781315370279.

[76] Genz, A. & Bretz, F. *Computation of Multivariate Normal and t Probabilities*. Lecture Notes in Statistics (Springer, Berlin, Heidelberg, Germany, 2009), 1st edn. doi:10.1007/978-3-642-01689-9.

[77] Pinheiro, J. C. & Bates, D. M. *Mixed-Effects Models in S and S-PLUS* (Springer, New York, USA, 2000), 1st edn. doi:10.1007/b98882.

[78] Pinheiro, J. C., Bates, D. M. & R Core Team. **nlme**: Linear and nonlinear mixed effects models (2022). R package version 3.1-164; https://CRAN.R-project.org/package=nlme.

[79] Müller, K. & Wickham, H. **pillar**: Coloured formatting for columns (2021). R package version 1.9.0; https://CRAN.R-project.org/package=pillar.

[80] Hijmans, R. J. **raster**: Geographic data analysis and modeling (2021). R package version 3.6-26; https://CRAN.R-project.org/package=raster.

[81] Bivand, R. & Rundel, C. **rgeos**: Interface to geometry engine - open source ('GEOS') (2022). R package version 0.6-4; https://CRAN.R-project.org/package=rgeos.

[82] Bivand, R., Keitt, T. & Rowlingson, B. **rgdal**: Bindings for the 'geospatial' data abstraction library (2022). R package version 1.6-7; https://CRAN.R-project.org/package=rgdal.

[83] South, A. **rnaturalearth**: World map data from natural earth (2017). R package version 1.0.1; https://CRAN.R-project.org/package=rnaturalearth.

[84] South, A. **rnaturalearthdata**: World vector map data from natural earth used in 'rnaturalearth' (2017). R package version 1.0.0; https://CRAN.R-project.org/package=rnaturalearthdata.

[85] South, A. **rnaturalearthhires**: High resolution world vector map data from natural earth used in **rnaturalearth** (2023). R package version 0.2.0; https://docs.ropensci.org/rnaturalearthhires.

[86] Wickham, H. & Seidel, D. **scales**: Scale functions for visualization (2020). R package version 1.3.0; https://CRAN.R-project.org/package=scales.

[87] Jordan, A., Krüger, F. & Lerch, S. Evaluating probabilistic forecasts with **scoringRules**. *Journal of Statistical Software* **90**, 1–37 (2019). doi:10.18637/jss.v090.i12.

[88] Pebesma, E. J. Simple features for R: Standardized support for spatial vector data. *The R Journal* **10**, 439–446 (2018). doi:10.32614/RJ-2018-009.

[89] Pebesma, E. & Bivand, R. *Spatial Data Science: With Applications in R* (Chapman & Hall/CRC, New York, USA, 2023), 1st edn. doi:10.1201/9780429459016.

[90] Strimas-Mackey, M. **smoothr**: Smooth and tidy spatial features (2021). R package version 1.0.1; https://CRAN.R-project.org/package=smoothr.

[91] Pebesma, E. J. & Bivand, R. S. Classes and methods for spatial data in R. *R News* **5**, 9–13 (2005). https://CRAN.R-project.org/doc/Rnews/.

[92] Bivand, R. S., Pebesma, E. J. & Gómez-Rubio, V. *Applied Spatial Data Analysis with R*. Use R! (Springer, New York, USA, 2013), 2nd edn. https://asdar-book.org/, doi:10.1007/978-1-4614-7618-4.

[93] Seiler, J. *et al.* Custom computer code for 'Co-occurrence patterns of malnutrition indicators among children in sub-Saharan Africa' (2025). doi:10.5281/zenodo.18087416.
