# Supplementary material for: Co-occurrence patterns of malnutrition indicators among children in sub-Saharan Africa
Source: Commun Med (Lond). 2026 Apr 9;6:195. doi: 10.1038/s43856-026-01426-8 (PMC13066028; doi:10.1038/s43856-026-01426-8)
Supplement: Supplementary file 3 — Description of Additional Supplementary Files [file 43856_2026_1426_MOESM3_ESM.pdf]

Description of Additional Supplementary Files

- File Name: *Supplementary Data 1*
  The data to reproduce Figure 1 using the R-script figures.R included in the Zenodo repository: https://doi.org/10.5281/zenodo.18087416.

- File Name: *Supplementary Data 2*
  The data to reproduce Figure 2 and 3 using the R-script figures.R included in the Zenodo repository: https://doi.org/10.5281/zenodo.18087416.

- File Name: *Supplementary Data 3*
  The data to reproduce Figure 4 and 5 using the R-script figures.R included in the Zenodo repository: https://doi.org/10.5281/zenodo.18087416.
